# Oral prednisolone for acute otitis media in children: a pilot, pragmatic, randomised, open-label, controlled study (OPAL study)

**DOI:** 10.1186/s40814-020-00671-5

**Published:** 2020-08-29

**Authors:** Respati W. Ranakusuma, Amanda R. McCullough, Eka D. Safitri, Yupitri Pitoyo, Widyaningsih Widyaningsih, Christopher B. Del Mar, Elaine M. Beller

**Affiliations:** 1 Institute for Evidence-Based Healthcare, Bond University, 14 University Drive, Robina, QLD 4226 Australia; 2 Clinical Epidemiology and Evidence-Based Medicine Unit, Dr. Cipto Mangunkusumo General Hospital – Faculty of Medicine Universitas Indonesia, Diponegoro 71, Jakarta, 10430 Indonesia

**Keywords:** Otitis media, Acute disease, Anti-bacterial agents, Glucocorticoids, Corticosteroids, Middle ear effusion, Pilot projects, Acoustic impedance tests

## Abstract

**Background:**

Acute otitis media (AOM) is associated with high antibiotic prescribing rates. Antibiotics are somewhat effective in improving pain and middle ear effusion (MEE); however, they have unfavourable effects. Alternative treatments, such as corticosteroids as anti-inflammatory agents, are needed. Evidence for the efficacy of these remains inconclusive. We conducted a pilot study to test feasibility of a proposed large-scale randomised controlled trial (RCT) to assess the efficacy of corticosteroids for AOM.

**Methods:**

We conducted a pilot, pragmatic, parallel, open-label RCT of oral corticosteroids for paediatric AOM in primary and secondary/tertiary care centres in Indonesia. Children aged 6 months–12 years with AOM were randomised to either prednisolone or control (1:1). Physicians were blinded to allocation. Our objectives were to test the feasibility of our full RCT procedures and design, and assess the mechanistic effect of corticosteroids, using tympanometry, in suppressing middle ear inflammation by reducing MEE.

**Results:**

We screened 512 children; 62 (38%) of 161 eligible children were randomised and 60 were analysed for the primary clinical outcome. All study procedures were completed successfully by healthcare personnel and parents/caregivers, despite time constraints and high workload. All eligible, consenting children were appropriately randomised. One child did not take the medication and four received additional oral corticosteroids. Our revised sample size calculation verified 444 children are needed for the full RCT. Oral corticosteroids did not have any discernible effects on MEE resolution and duration. There was no correlation between pain or other symptoms and MEE change. However, prednisolone may reduce pain intensity at day 3 (Visual Analogue Scale mean difference − 7.4 mm, 95% confidence interval (CI) − 13.4 to − 1.3, *p* = 0.018), but cause drowsiness (relative risk (RR) 1.8, 95% CI 1.1 to 2.8, *p* = 0.016). Tympanometry curves at day 7 may be improved (RR 1.8, 95% CI 1.0 to 2.9). We cannot yet confirm these as effects of corticosteroids due to insufficient sample size in this pilot study.

**Conclusions:**

It is feasible to conduct a large, pragmatic RCT of corticosteroids for paediatric AOM in Indonesia. Although oral corticosteroids may reduce pain and improve tympanometry curves, it requires an adequately powered clinical trial to confirm this.

**Trial registration:**

Study registry number: ACTRN12618000049279. Name of registry: the Australian New Zealand Clinical Trials Registry (ANZCTR). Date of registration: 16 January 2018.

## Background

Antibiotic resistance is a global health threat, largely because of antibiotic use [1–3]. Use of antibiotics is also associated with unfavourable effects (e.g. vomiting, diarrhoea) [4]. There is a particularly high rate of antibiotic prescribing for acute respiratory infections (ARIs) in outpatient settings (primary and secondary care): 50% in the USA; 53% in European countries; 34% in Malaysia; and up to 78% in Indonesia [5–8].

Acute otitis media (AOM), a middle ear inflammation, is an ARI that is commonly found in children, particularly before the age of five [9, 10]. Antibiotics are commonly prescribed. A Cochrane review showed that antibiotics are effective in improving acute pain and tympanometry results, as well as other clinical outcomes (e.g. tympanic membrane perforation, contralateral AOM). However, due to their modest benefits along with significant potential for unfavourable effects, antibiotics are not mandatory treatment for AOM, particularly for mild AOM. Therefore, it is important to emphasize both benefits and harm of antibiotic use to the patients and their parents and involve them in treatment decision making in the management of AOM.

In Australia, 89% of new AOM cases were managed by antibiotics between 2010 and 2015 [11]. In Indonesia, our feasibility survey showed that up to 88% of physicians would prescribe antibiotics for mild AOM, although antibiotics are most beneficial for severe AOM (i.e. severe symptoms, young children with bilateral AOM, tympanic membrane perforation) [9]. These rates are higher compared to other western countries such as the USA (57.6%), Iceland (70.4%), Denmark (73.7%) and Sweden (86.7%) [12–14]. This condition may be affected by two contradictory Indonesian practice guidelines for AOM [15, 16]. The AOM guideline for primary care practitioners recommends the use of antibiotics for both mild and severe AOM. The only difference is the antibiotic dose, which recommends a higher dose for severe AOM [15]. On the other hand, the guideline for Ear, Nose and Throat (ENT) specialists recommends antibiotic use for only severe AOM, although it does not specifically describe the definition of severe AOM [16]. In addition, despite of the existence of national regulation on antibiotic use, its implementation is less enforced in Indonesia and leads to antibiotic self-medication [17, 18].

High rates of antibiotic use, despite of its modest benefits and side effects and antibiotic resistance, indicate the necessity to find effective alternative treatments for AOM (e.g. decongestants, herbal preparations, corticosteroids) [19–21]. The existing alternative treatments have insufficient evidence to be recommended in clinical practice [19–21]. Since inflammation is a key mechanism of AOM, corticosteroids are a potential treatment. Although they have been effectively used for other ARIs (e.g. pneumonia, bacterial meningitis) [22, 23], their effect on AOM remains unclear [24, 25]. Aside from potential beneficial effects on inflammation, corticosteroids also may cause side effects. Gastrointestinal disturbances and behavioural changes have been identified as common side effects [26]. Although short-term use of a corticosteroid is unlikely to induce serious harm, one systematic review reported 1% of children experienced increased susceptibility to infections due to the immunosuppression effect of corticosteroids [26].

Our Cochrane review shows that systemic corticosteroids may be effective in improving clinical symptoms of AOM [24]. However, this was based on one small study demonstrating uncertain effects of systemic corticosteroids for AOM due to a wide confidence interval that included both benefits and harm. High-quality evidence is crucial to address uncertainties around the effects of corticosteroids for AOM. Therefore, we plan to conduct an adequately powered clinical trial to assess both benefits and harm of corticosteroids for children with AOM. Our original sample size calculation demonstrated that we need 760 children to be able to detect actual effects of oral corticosteroids for children with AOM. Prior to this, we conducted a pilot study to test the feasibility of characteristics of our full RCT design and procedures in 60 children, including a mechanistic sub-study using tympanometry (tympanometry sub-study) to study the effect of corticosteroids on middle ear effusion (MEE). The clinical findings from this study, although they cannot be definitive due to a small sample size, can indicate the direction of potential clinical effects of oral corticosteroids for AOM, which can then be confirmed in an adequately powered clinical trial.

## Methods

### Study aims and objectives

This pilot study aimed to test all pre-specified methods and procedures that are planned to be implemented in the full RCT, but in a smaller sized study.

The objectives for the pilot study were to assess the feasibility of characteristics of the full RCT design and procedures (e.g. recruitment, randomisation, outcome measurement, experience and obstacles of physicians and parents/caregivers, sample size verification) and assess the mechanistic effect of corticosteroids in suppressing inflammation in the middle ear specifically by reducing MEE.

Prior to our study implementation, our study protocol was reviewed and approved by the Ethics Committee Faculty of Medicine Universitas Indonesia and the Bond University Human Research Ethics Committee (BUHREC) Australia (see Declarations section).

### Study design and setting

This was a pilot parallel, pragmatic, stratified, randomised, open-label, single-blind, controlled study in an allocation ratio of 1:1. We planned to conduct this study in seven hospitals in Jakarta and Bekasi as described in our protocol [27]; however, we only received research permits from six hospitals. Due to low rate of recruitment, we added two primary care centres in Jakarta to the study.

### Participants

#### Inclusion criteria

We included children aged 6 months to 12 years old with AOM, defined as current onset (within 48 h) of AOM-relevant symptoms (e.g. earache, ear tugging/rubbing or irritability in non-verbal children). Otoscopic findings of acute inflammation (e.g. erythema) and middle ear effusion (e.g. bulging, air-fluid level) confirmed the diagnosis.

#### Exclusion criteria

We excluded children (1) with major and severe medical conditions (e.g. heart diseases, tuberculosis), (2) who were immunocompromised (e.g. HIV infection, under cancer treatment), (3) with congenital malformations and/or syndromes (e.g. cleft palate, Down syndrome), (4) with high risk of strongyloidiasis infections, (5) with ear ventilation tube(s), (6) who had been exposed to persons with varicella or active Zoster infection in the preceding 3 weeks without prior varicella immunisation or infection, (7) who had taken systemic (oral, injection) or topical steroids in the preceding 4 weeks, (8) who had taken antibiotics in the preceding 2 weeks and (9) who were hypersensitive to prednisolone or prednisone, or other corticosteroids. Further details of the recruitment process including obtaining consent are in our protocol [27].

#### Study intervention arm

**Fig. 1:**
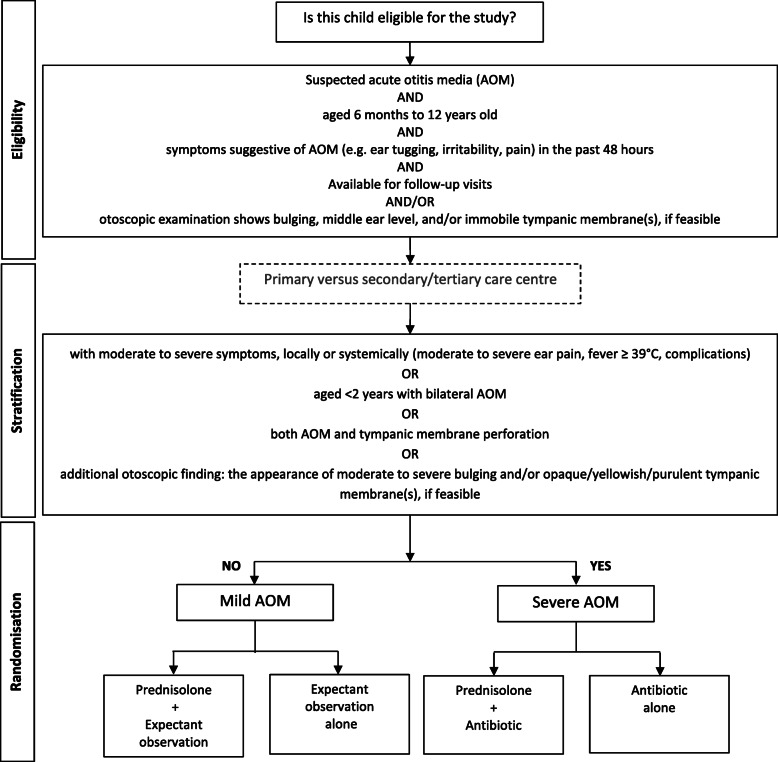
Flowchart of the study stratification and randomisation

We gave prednisolone tablets (Lupred®5) at a daily dose of 1–2 mg/kg of body weight for 5 days, which was operationalised based on age: 10 mg/day for children aged 6 months to up to 2 years; 20 mg/day for children aged 2 up to 6 years; and 30 mg/day for children aged 6 to 12 years [28]. We provided liquid sweetener to make prednisolone more palatable. Children with mild AOM who were randomised to the intervention arm received prednisolone plus expectant observation, whilst those with severe AOM received prednisolone plus antibiotics (see Fig. 1). Further details of administration and timing of study medication are described in our protocol [27].

#### Control arm

Children with mild AOM who were randomised to the control arm received expectant observation alone, whilst those with severe AOM received antibiotics alone (see Fig. 1).

#### Concurrent treatment

Physicians were able to prescribe medications for symptoms (e.g. analgesic, decongestant) as per their usual practice. The physicians were not able to prescribe oral corticosteroids.

### Outcomes

For the first objective of assessing the feasibility of characteristics of our full RCT design and procedures, we measured six outcomes: (1) recruitment rates; (2) successful completion of the study procedures; (3) successful measurement of planned outcomes; (4) the experiences and barriers of participating healthcare personnel (i.e. physicians, nurses, audiologists, pharmacists) and parents/caregivers in measuring planned outcomes; (5) adherence to study visits and study medication; and (6) verification of the sample size calculation for the full RCT using pain measures in the control group.

For the second objective of assessing the mechanistic effect of corticosteroids in suppressing inflammation in the middle ear, we measured three outcomes: (a) change in MEE at various time points; (b) duration of MEE; and (c) correlation between ear pain and other symptoms with the changes in MEE at various time points.

Recruitment rate was defined as the proportion of consultations with potentially eligible children who provided their consent to be included in the study. We assessed this outcome monthly using a logbook completed by participating nurses and physicians.

The successful completion of the study procedures and outcome measures was defined as the proportion of participating healthcare personnel and parents/caregivers who were able to conduct study procedures and measure outcomes in order to obtain valid results. The study procedures were (1) obtaining consent; (2) recruitment and stratification; (3) otoscopic assessment; (4) pain measures using Visual Analogue Scale (VAS) and Acute Otitis Media Severity of Symptoms (AOM-SOS) scale; (5) randomisation, treatment allocation and prescription dispensing; (6) preparation, completion, compilation and storage of case report forms (CRFs); (7) tympanometry examination and interpretation; (8) preparation and dispensing of study medication; and (9) completion of the symptom diary. These outcomes were measured using the logbook, case report form and the symptom diary (Additional file 1. Case report forms).

For the assessment of the experience and barriers to measuring outcomes, we used a feedback form (see Additional file 1. Case report forms). Each question represented a study procedure and had five responses (‘very easy’, ‘easy’, ‘neutral’, ‘difficult’ and ‘very difficult’). We asked the healthcare personnel and parents to choose one response. We asked those who chose ‘difficult’ to choose obstacle(s) describing their experience during that procedure or measurement. They could choose more than one option or write their personal experience.

We assessed the adherence to study medication by identifying the proportion of children who took the study medication (prednisolone) for 5 days per protocol divided by all children in the prednisolone group. This outcome was measured using the symptom diary. The adherence to the study visit was assessed by identifying the proportion of children who came to follow-up visits at day 3, day 7, day 30, and day 90 per protocol divided by all children randomised to the study. This outcome was measured using the follow-up card, the consent form and the symptom diary.

The verification of sample size for the full RCT was calculated by identifying the proportion of children with mild and severe AOM in the pilot study with ongoing pain represented as VAS score ≥ 5 mm, at day 3, in the control group.

We assessed change in MEE at day 3, day 7, day 30 and day 90 by measuring static acoustic admittance (SAA), which is defined as ‘the amount of energy absorbed by the tympanic membrane and middle ear’. We assumed that a difference of 0.3 mmho was a minimum clinically important one for SAA [29].

For duration of MEE, we only conducted tympanometry at follow-up visits. We were not able to identify whether the effusion had persisted, disappeared or reappeared between these visits. Therefore, we reported this as the proportion of children who had a complete resolution of MEE which is represented by a type A curve at follow-up visits. We used the modification of Jerger to classify the tympanogram curve types, as follows: (1) a type A curve indicating a normal middle ear; (2) type C curves including a C1 curve, which indicates a transition from a normal middle ear to an early MEE, and a C2 curve representing an early MEE; and (3) a type B curve, strongly indicating the presence of MEE or a definite MEE [30–33].

We assessed the correlation between ear pain and other symptoms (i.e. ear tugging, irritability, crying, lack of sleep, lack of appetite, loss of playfulness, fever) using VAS and AOM-SOS assessed by parents in the symptom diary with the changes in SAA at day 3, day 7, day 30 and day 90.

The planned primary outcome of the full RCT is the proportion of children with pain that has not reduced by the minimum clinically important amount (10 mm VAS) by day 3 [34, 35]. The secondary outcomes are (1) the proportion of children with ongoing pain (VAS ≥ 5 mm) [36] at day 1, day 3, day 5, day 7 and day 14; (2) reduction of pain intensity measured using VAS at similar time points; (3) reduction of overall symptoms measured using AOM-SOS at similar time points [37]; (4) reduction of overall pain duration; (5) complications related to AOM; (6) the proportion of children with mild AOM requiring antibiotics and those with severe AOM requiring second-line antibiotics; (7) AOM recurrence; (8) adverse effects; and (9) the adherence to study medication. For the VAS and AOM-SOS assessment, we used data from the symptom diary completed by the parents. As this is a pilot, we do not report here the primary outcome of the full RCT. However, we do report the first secondary outcome. Further details of data collection for the pilot and full RCT are described in our protocol paper [27].

### Sample size

We did not formally determine the sample size for this pilot study. We included 60 children with AOM in our pilot study based on sample size calculation for the tympanometry sub-study [27]. Our feasibility survey demonstrated that it would require 9 months including 3-month follow-up to recruit 60 children. We did not conduct an interim analysis due to the small sample size and short study duration.

#### Recruitment and stratification

For the full RCT, we plan to stratify children by clinical specialty or healthcare centre level (primary care or secondary/tertiary healthcare centres) and severity of AOM (mild or severe). As we intended to only include ENT specialists who worked at secondary/tertiary centres in this pilot study, we stratified children based only on AOM severity. Details on stratification criteria are in the protocol paper [27].

To help the recruitment and stratification process, including randomisation and tympanometry examination where necessary, we recruited and trained seven research assistants, including one administrative assistant, to support the implementation of the study (RO, DN, IG, RA, RS, FR, VV).

#### Randomisation and allocation concealment

After confirmation of eligibility, and completion of the clinical examination, but prior to randomisation, physicians dispensed two prescriptions to the nurse. The first prescription was for general AOM medications (e.g. antibiotics, decongestants). The second one was for the study medication. The nurse then only dispensed the second prescription to patients who were randomised to the intervention arm. Further details of randomisation process, including the preparation, dispensing and central storage of study medication are in the protocol paper [27].

#### Blinding

The appointed nurses and patients or parents/caregivers were aware of the intervention allocation. The physicians and audiologists were blinded to the allocation [27]. At the primary care centre, the principal investigator (RR) and research assistants (RO, DN, IG, RA, RS, FR) conducted randomisation and tympanometry examination because of the limited availability of healthcare personnel for the study. Therefore, it was not possible to conceal allocation after randomisation. However, the research assistants were not involved in the treatment decisions and ensured the clinician was not aware of the intervention allocation.

#### Statistical methods

We report the outcomes of the pilot study (e.g. recruitment rate, the success and ability of study procedures and measures, adherence to study visits and medication) as proportions in percentages (*n*, %).

For the verification of sample size calculation, we used the proportion of the children in both mild and severe groups, and pain measures among the controls to update our calculation of failure rate of ongoing pain at day 3. We did not change the assumed relative of risk on ongoing pain at day 3, as the pilot results arise from a small sample.

For the clinical outcomes, since it was a pilot study and we did not want to reveal the result of the primary outcome of the proposed full RCT, we only report the proportion of children with ongoing pain (binary outcome) and reduction of pain and other symptom severity (continuous outcome) at day 1, day 3, day 5, day 7 and day 14. For clinical and tympanometry continuous outcomes (i.e. pain and symptoms scores measured by VAS and AOM-SOS, SAA value), we conducted linear regression to determine the unadjusted and adjusted mean difference (MD), 95% confidence interval (CI) and the *p* values for the comparison between groups at day 3 as a primary time point . The adjusted MD used the baseline result (equivalent to ANCOVA). Spearman’s correlation coefficient was calculated to determine the correlation between ear pain using VAS and SAA values on the affected ear for unilateral AOM and the worst ear for bilateral AOM, as well as between other relevant symptoms using AOM-SOS and the similar SAA values. We used STATA 15.1 software for statistical analysis.

For clinical and tympanometry binary outcomes (i.e. the proportion of children with persisting pain of VAS ≥ 5 mm and those who had complete MEE resolution at various time points), we conducted chi-squared tests and reported relative risk (RR) and 95% CI for the comparison between groups. We presented the *p* values of the outcomes measured at day 3 (primary time point). We used Fisher’s exact test to determine statistically significant differences in effect estimates of binary outcomes between the prednisolone and control group where there were small event numbers (< 10 per variable cell).

For time to pain resolution, we identified the time point where children had resolution of pain (VAS < 5 mm) and presented these as a median, and then compared the median between the two groups using a Wilcoxon rank sum test.

## Results

### Recruitment

**Fig. 2:**
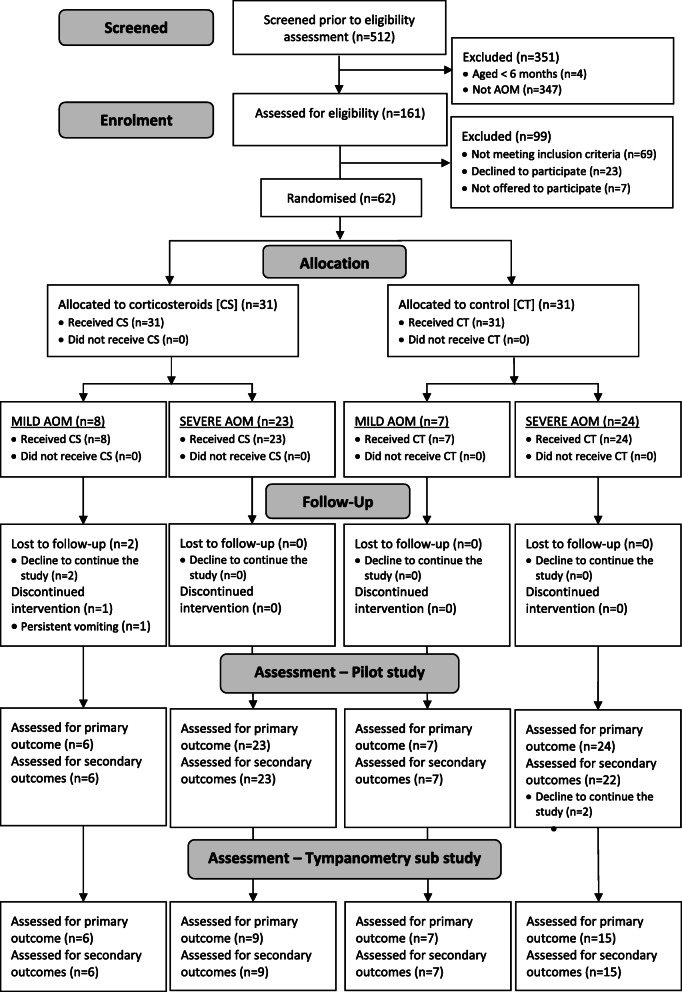
Study flowchart

We screened 512 children with ear pain (22 February–30 November 2018) and 161 children (31%) were assessed using the eligibility criteria (see Fig. 2). All physicians were able to confirm AOM using an otoscope. Sixty-two (38%) eligible children were stratified to mild and severe AOM. Thirty-one children were randomly allocated to the prednisolone group (8 mild and 23 severe AOM) and 31 children to the control group (7 mild and 24 severe AOM). Two children left the study before data collection at day 3 resulting in 60 children (29 prednisolone versus 31 control group) being analysed for the planned primary outcome of the full RCT. The study was ended on February 2019 allowing for the 3-month follow-up of the last patient.

### Baseline data

The baseline characteristics between the two groups were similar in gender and had an age range of 61 to 73 months (see Table 1). There were more children in the control group whose parents had a low education level (primary and secondary education), were exposed to parental smoking (passive smoking) or received amoxicillin and amoxicillin/clavulanate and acetaminophen at baseline. Children in the prednisolone group were more likely to receive cefixime. Forty-seven percent of the children were found to have hyperaemic tympanic membrane only on the otoscopic examination.

**Table 1:** Baseline and clinical characteristics of randomised children by treatment group

Characteristics	Prednisolone (*n* = 31)	Control (*n* = 31)
Baseline characteristics		
Age (months) mean ± SD	60. 7 ± 32.2	73.2 ± 38.3
Sex—male (*n*; %)	15 (48)	18 (58)
Breastfeeding (*n*; %)	28 (90)	27 (87)
Breastfeeding until at least the first 6 months of life (n; %)	20 (71)	19 (70)
Child day care attendance (*n*; %)	1 (3)	1 (3)
Duration per week (h) mean ± SD	50	35
Pre-school or school attendance (*n*; %)	21 (68)	20 (64)
Duration per week (hours) mean ± SD	4.9 ± 2.0	4.7 ± 2.0
Parental education (father)^a^		
Primary education (i.e. elementary school)	1 (3)	2 (6)
Secondary education (i.e. middle and high school)	13 (42)	17 (55)
Tertiary education (e.g. diploma, bachelor, masters)	16 (52)	11 (35)
Parental education (mother)^a^		
Primary education (i.e. elementary school)	0 (0)	2 (6)
Secondary education (i.e. middle and high school)	12 (39)	17 (55)
Tertiary education (e.g. diploma, bachelor, masters)	19 (61)	11 (35)
Pneumococcal vaccinations (*n*; %)	9 (29)	7 (23)
Influenzae vaccinations (*n*; %)	6 (19)	7 (23)
≥ 3 episodes of acute respiratory infections in the past year (n; %)	23 (74)	22 (71)
First episode of AOM	20 (64)	21 (68)
First episode of AOM at ≤ 2 years of age (*n*; %)	8 (26)	3 (10)
≥ 3 episodes of ear infection in the past year (*n*; %)	3 (10)	2 (6)
> 3 children in one house (*n*; %)	0 (0)	0 (0)
Passive smoking (*n*; %)	14 (45)	20 (64)
Ear discharge (*n*; %)	11 (35)	8 (26)
Concomitant diseases (*n*; %)		
Allergic rhinitis	3 (10)	2 (6)
Bronchial asthma	0 (0)	1 (3)
History of atopy in the family	12 (39)	9 (29)
AOM lateralisation—unilateral (*n*; %)	20 (64)	18 (58)
Clinical characteristics		
Common cold	27 (87)	28 (90)
Nose abnormalities (e.g. oedema, discharge)	23 (85)	23 (82)
Tonsil abnormalities (e.g. hyperaemic, oedema)	15 (55)	15 (53)
Throat abnormalities (e.g. hyperaemic, oedema)	15 (55)	8 (28)
Diagnosis of AOM		
Confirmed by otoscope		
Hyperaemic tympanic membrane only	12 (39)	17 (55)
Hyperaemic tympanic membrane and other signs of inflammation/middle ear effusion^b^	23 (74)	21 (68)
Confirmed by otoscope and clarified by tympanometry^c^	25 (86)	25 (86)
Initial antibiotic given (*n*; %)		
Amoxicillin	4 (13)	11 (35)
Amoxicillin/clavulanate	5 (16)	8 (26)
Cefixime	12 (39)	3 (10)
Cefadroxil	1 (3)	1 (3)
Trimethoprim/sulfamethoxazole	0 (0)	1 (3)
Clarithromycin	1 (3)	0 (0)
Other treatment given by doctors at initial visit (*n*; %)		
Acetaminophen	9 (29)	16 (52)
Nonsteroidal anti-inflammatory drugs	4 (13)	5 (16)
Decongestants and/or antihistamine	26 (84)	22 (71)
Cough medicine	18 (58)	14 (45)
Antibiotic ear drops	9 (29)	6 (19)
Nasal (topical) decongestant	6 (19)	2 (6)
Nasal corticosteroid	0 (0)	1 (3)
Vitamins or herbals	3 (10)	8 (26)
Ear diathermy	0 (0)	1 (3)
Inhalation	0 (0)	1 (3)
Others (e.g. mefenamic acid, nasal douching)	3 (10)	5 (16)
Tympanometry test (*n*; %)	29 (93)	29 (93)
Complete	15 (52)	18 (62)
Partial completion	0 (0)	6 (21)
Sufficient values for analysis	15 (52)	22 (76)
Type A	4 (27)	6 (27)
Type C1	2 (13)	4 (18)
Type C2	1 (7)	1 (4)
Type B (or flat)	8 (53)	11 (50)

^a^We could not obtain the information of father’s (*n* = 2) and the mother’s education level (*n* = 1)

^b^A child with bilateral AOM may have two different otoscopic results (e.g. hyperaemic tympanic membrane only and hyperaemic tympanic membrane with other signs of inflammation/middle ear effusion)

^c^Four patients did not undergo tympanometry examination due to severe pain, not recommended by physicians due severe bulging, uncooperative child and nurse forgot. Patients with tympanic membrane perforation were considered as confirmed by otoscope and tympanometry

### Numbers analysed

For the primary outcomes of the full RCT, we analysed 60 children at day 3. For the secondary outcomes, we analysed 58 children (29 prednisolone versus 29 control group) as two more children left the study after data collection at day 3. For the tympanometry sub-study, we conducted tympanometry examinations on 58 children (93%) and analysed 37 children (15 prednisolone versus 22 control group) who had sufficient tympanometry findings at both baseline and day 3. We were not able to fulfil the sample size to 60 children due to difficulty in recruitment and budget constraints which prevented the extension of the recruitment period.

### Outcomes and estimation

#### Recruitment rate

We collected logbooks from each study site to measure the recruitment rate. However, most nurses did not complete the logbook appropriately. The average recruitment rate was 38.5% of potentially eligible children (see Table 2). The main reasons for exclusion were (a) onset > 2 days (24%); (b) lack of interest or reluctance of parents to participate (14%); and (c) prior intake of antibiotics/steroids (10%). Three sites did not contribute to patient recruitment because of no eligible cases. Only one private hospital contributed to the study recruitment. Therefore, we added two primary care centres to the study.

**Table 2:** Recruitment rates

Recruitment details according logbook	Feb	Mar	Apr	May	Jun	Jul	Aug	Sep	Oct	Nov	Monthly mean
Recruitment rate^a^ (%)	0	48	33.3	44.4	33.3	66.7	40	40	35.7	20.7	38.5
Screened children with AOM	1	25	18	9	6	15	15	15	28	29	16.1
Recruited to the study	0	12	6	4	2	10	6	6	10	6	6.2
Did not meet study criteria	0	7	4	2	2	4	7	8	14	21	
Onset > 2 days	0	4	3	2	1	3	4	4	7	10	
Prior antibiotic/steroid intake	0	3	1	0	0	1	1	2	4	8	
Chronic/immunodeficiency disorders	0	0	0	0	0	0	2	0	0	0	
Unable for follow-up visits	0	0	0	0	1	0	0	2	3	3	
Declined to participate	1	5	6	3	1	1	0	1	4	1	
Not offered participation	0	1	2	0	1	0	2	0	0	1	

^a^Recruitment rate = children with AOM aged six month to 12 years who were recruited into the study divided by all children with AOM aged six months to 12 years who were being assessed for the study by participating physicians

#### The successful completion of the study procedures and outcome measures

Prior to the study, we provided training to 66 physicians (i.e. ENT specialists, GPs, paediatricians), 39 nurses, 35 pharmacists and 6 audiologists. During the training, we coached and assisted them in conducting all study procedures and measures per protocol.

Twenty-three of 85 parents/caregivers of eligible children (27%) declined to participate in the study (see Table 2). All physicians (100%) successfully recruited and stratified eligible children based on their AOM severity, performed an otoscopic assessment and measured pain and other relevant symptoms using VAS and AOM-SOS, and reported the findings as self-reporting assessment in the CRF. All eligible children were successfully randomised and allocated to their randomised intervention by nurses/research assistants. All patients had the study medication prescriptions dispensed as per protocol. All nurses were able to appropriately prepare, compile and store the CRF. All audiologists successfully performed tympanometry examination. However, there were incomplete values caused by lack of sensitivity of tympanometry. One of 31 study medication packages (3%) was not dispensed by the pharmacist, which had to be home delivered by the researcher (RR).

One hundred percent of symptom diary data was completed for analysis. However, only 60% of parents/caregivers were able to complete the symptom diary per protocol, which required us to collect data and clarify unclear responses by interviewing 25 parents retrospectively (see Table 3). We regularly checked the completion of the symptom diary of day 0 to 3 at the first follow-up visit (day 3) and day 4 to 7 at the second visit (day 7), and after the diary collection at day 14. We expected this strategy may reduce recall bias. We interviewed the parents directly during the consultation at the follow-up visits and at the follow-up by phone at day 14.

**Table 3:** The adherence to the study

Adherence to the study	Prednisolone (*n* = 31)	Control (*n* = 31)
Not compliant to the completion of symptom diary		
No data after baseline for primary outcome analysis	2	0
Data collected retrospectively by interview	4	13
Unclear responses clarified by interview	5	3
Not compliant to study medication^a^		
Missed one dose, but taken on the next day	1	–
Vomited constantly and stopped the study medication	1	–
Took half of dose, vomited < 30 min and took another half of dose	1	–
Took medicine in the afternoon (not in the morning)	2	–
Not compliant to the follow-up visits		
Delayed timing of follow-up visit	3	3
Left study	2	2
Additional interventions		
Received additional oral corticosteroids	3^b^	1^c^
Received intervention of co-medication from study investigator	2	5

^a^Nine patients did not complete diary, but the adherence confirmed by interview

^b^At day 10, 12 and 60

^c^At day 7

#### Experiences and barriers to measuring planned outcomes of the full RCT

We measured this outcome using a feedback form. We only invited physicians and nurses who recruited patients or were involved in data collection for at least two patients to provide feedback. We obtained feedback from 15 ENT specialists (15/51; 29%), six GPs (6/9; 67%), 16 nurses (16/39; 41%), six pharmacists (6/35; 17%) and four audiologists (4/6; 67%).

Most ENT specialists and GPs rated obtaining consent from parents, recruiting and stratifying patients into the study, and using otoscopes as ‘easy’. The common obstacles were (1) reluctance to participate due to the term ‘research’ in the consent form; (2) lengthy time to deliver extensive study information; (3) time constraints due to the need for an increased appointment length and pressure of patient numbers; and (4) a complex eligibility form. General practitioners found using an otoscope was challenging in some patients due to the narrowness of ear canals and uncooperative children. Most ENT specialists (73%) rated completing the CRFs as ‘easy’, whilst most GPs rated this as ‘neutral’ (50%). The ENT specialists recommended simplification of the CRFs.

Most ENT specialists and GPs rated VAS (71% and 75%, respectively) and AOM-SOS (85% and 100%) as ‘easy’, although more information on pain description was required. They suggested using a facial scale to measure pain. They found AOM-SOS was suitable to assess pain in young children.

Most nurses rated the randomisation process and CRF compilation, preparation and storing as ‘easy’, and rated treatment allocation and prescription dispensing as ‘neutral’. They found obstacles when accessing the randomisation website due to complicated access steps and unstable internet connection. They also found these were time-consuming, particularly in terms of randomisation and CRF compilation, which were not always feasible due to workload. Most nurses contacted the 24-h call centre for assistance in the randomisation and recruitment process.

The pharmacists and audiologists rated the preparation and dispensing of study medication, and tympanometry examination as ‘very easy’ to ‘easy’. We also obtained feedback from our research assistants (*n* = 6) who conducted tympanometry examination in primary care centre. Only one rated this as ‘difficult’ due to the narrowness of ear canals.

**Fig. 3:**
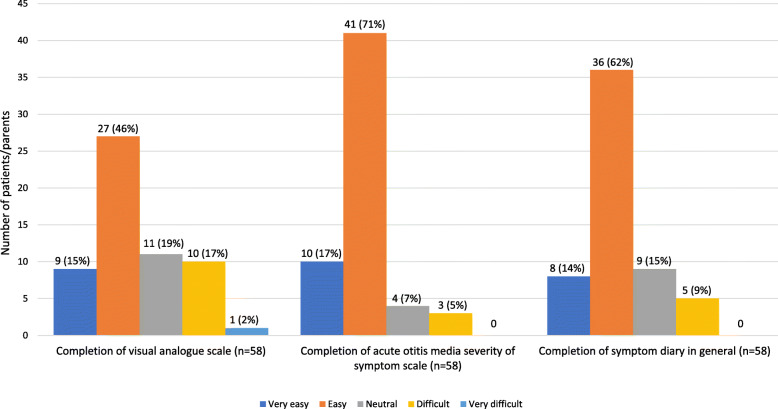
Parents’ experience in measuring planned outcomes for the main study

We obtained feedback from parents regarding the completion of the symptom diary (see Fig. 3). The majority of parents rated VAS (47%), AOM-SOS (71%) and the completion of symptom diary (62%) as ‘easy’. Parents preferred to use a numeric pain scale.

#### Adherence to study visits and study medication

Fifty-eight children completed all follow-up visits and four children left the study (see Table 3). We visited homes of those who were not able to come for their follow-up visits. During this visit, we did not prescribe any medication and recommended they visit the hospital/primary care centre for any concerning condition (e.g. worsening AOM, complications).

Four children received additional oral corticosteroids: one from a participating physician for AOM and three children received it from other physicians for asthma, prolonged cough due to allergy and sore throat. All received this after measurement of the primary outcome (see Table 3).

#### Interference by research investigator

Due to the lack of available second-line antibiotics and other medications in primary care centres, the principal investigator (RR), who was not blinded to the intervention allocation, provided several medications to seven patients: second-line antibiotics (4/62; 6%); ibuprofen (4/62; 6%); combined decongestant-antihistamine (6/62; 10%); cough syrup (5/62; 8%); topical decongestant (4/62; 6%); and nasal saline drops (1/62; 2%). More children in the control group received interference, since most children in primary care centres were randomly allocated to this group by chance.

#### Verification of sample size calculation for the full RCT

Based on our original sample size calculation, we need to enrol 760 children with AOM. We estimated, based on data by Rovers et al. [38], that there would be 35% of the total sample of children with AOM in the severe group, of which 57.5% would have ongoing pain at day 3 in the control group. However, in our study, 78% of our total sample was in the severe group with risk of ongoing pain (≥ 5 mm VAS) at day 3 in control group of 42%. Of the children with mild AOM, 57% in the control group had ongoing pain at day 3. The average proportion of children with ongoing pain when combining the mild and severe groups was 45.2%. With our original assumption of 0.70 risk ratio with steroids, we will need to study 201 experimental and 201 control subjects to be able to reject the null hypothesis with probability (power) 0.8 and type I error probability of 0.05. We will use an uncorrected chi-squared statistic to evaluate this null hypothesis. With a 10% allowance for dropouts, the total sample size becomes 444.

There is a notable difference in the sample size estimation between our original sample size and our pilot study (see Table 4). The calculation of our original sample size was based on assumptions from a meta-analysis of studies conducted in developed countries. Our updated sample size using the pilot study results demonstrated that we need a smaller sample size for the full RCT if it is conducted in an urban setting in a developing country. The sample size may change if the study is conducted in different settings or countries. We are therefore presenting our assumptions for the sample size calculation for a clinical trial conducted in different settings (see Table 4).

**Table 4:** Sample size assumptions for a clinical trial of corticosteroids for AOM conducted in different settings

Proportion of children	Original assumption [38]^a^	Middle scenario	Pilot observed result^b^
With severe AOM	35%	56%	78%
With severe AOM AND ongoing pain	57.5%	50%	42%
With mild AOM	65%	43%	22%
With mild AOM AND ongoing pain	36%	46%	57%
With severe and mild AOM AND ongoing pain	31.6%	38.4%	45.2%
Sample size calculation^c^	760	570	444

^a^From a meta-analysis of studies conducted in developed countries

^b^Our pilot study was conducted in a developing country, urban setting

^c^The sample size includes a 10% allowance for dropouts

#### Change of middle ear effusion

We found no difference in middle ear effusion (MEE) change represented by SAA between the prednisolone and control groups at day 3 (MD 0.04 mmho, 95% − 0.07 to 0.16), day 7 (MD 0.07 mmho, 95% − 0.06 to 0.19), day 30 (MD − 0.05 mmho, 95% − 0.19 to 0.09) and day 90 (MD 0 mmho, 95% − 0.14 to 0.14). Consistent results were found after adjustment for the baseline results (see Table 5). All differences were well below the minimum clinical important difference of 0.3 mmho.

**Table 5:** Static acoustic admittance values in the affected (unilateral AOM) or the worst ear (bilateral AOM)

Static acoustic admittance: mmho mean ± SD	Prednisolone (*n* = 15)	Control (*n* = 22)	Unadjusted mean difference	*p* value	Adjusted mean difference^a^	*p* value
Day 0 (visit 1)	0.19 ± 0.13	0.24 ± 0.22	− 0.05 (− 0.18, 0.08)			
Day 3 (visit 1)	0.26 ± 0.15	0.22 ± 0.17	0.04 (− 0.07, 0.16)	0.43	0.07 (− 0.02, 0.16)	0.13
Day 7 (visit 2)	0.32 ± 0.15	0.25 ± 0.20	0.07 (− 0.06, 0.19)		0.08 (− 0.03, 0.20)	
Day 30 (visit 3)	0.32 ± 0.18	0.37 ± 0.22	− 0.05 (− 0.19, 0.09)		− 0.03 (− 0.16, 0.10)	
Day 90 (visit 4)	0.41 ± 0.18	0.41 ± 0.22	0 (− 0.14, 0.14)		0.02 (− 0.11, 0.16)	

^a^Adjusted for the baseline static acoustic admittance value

#### Duration of middle ear effusion

Although the confidence interval was very wide, there was no difference in the proportion of children who had complete resolution of MEE between the prednisolone and control groups at day 3 (RR 1.76, 95% CI 0.65 to 4.73), day 7 (RR 1.47, 95% CI 0.71 to 3.04), day 30 (RR 1.07, 95% CI 0.57 to 2.00) and day 90 (RR 1.17, 95% CI 0.80 to 1.72). We also identified any improvement of curve type from the baseline and previous results. Improvement was defined as an improvement from type B curve to type C2, C1 or A curve; or from type C2 curve to type C1 or A curve; or type C1 to A curve; or persisting type A curve. Table 6 shows the tympanometry curve improved compared to the baseline in more children in the prednisolone group at day 7 (RR 1.76, 95% CI 1.04 to 2.97).

**Table 6:** Tympanometry finding in the affected (unilateral AOM) or the worst ear (bilateral AOM)

Tympanometry findings	Prednisolone (*n* = 15)	Control (*n* = 22)	Effect estimate (relative risk)	*p* value
Complete middle ear effusion resolution^a^ (*n*, %)				
Day 3 (visit 1)	6 (40)	5 (23)	1.76 (0.65, 4.73)	0.29
Day 7 (visit 2)	8 (53)	8 (36)	1.47 (0.71, 3.04)	
Day 30 (visit 3)	8 (53)	11(50)	1.07 (0.57, 2.00)	
Day 90 (visit 4)	12 (80)	15 (68)	1.17 (0.80, 1.72)	
Improvement of curve type from baseline visit^b^ (*n*, %)				
Day 3 (visit 1)	7 (47)	7 (32)	1.47 (0.65, 3.32)	0.49
Day 7 (visit 2)	12 (80)	10 (45)	1.76 (1.04, 2.97)	
Day 30 (visit 3)	10 (67)	14 (64)	1.05 (0.65, 1.69)	
Day 90 (visit 4)	14 (93)	16 (73)	1.28 (0.96, 1.71)	
Improvement of curve type from previous visit^c^ (*n*, %)				
Day 3 (visit 1)	7 (47)	7 (32)	1.47 (0.65, 3.32)	0.49
Day 7 (visit 2)	9 (60)	9 (41)	1.47 (0.76, 2.81)	
Day 30 (visit 3)	9 (60)	13 (59)	1.01 (0.59, 1.74)	
Day 90 (visit 4)	13 (87)	15 (68)	1.27 (0.89, 1.80)	

^a^Complete resolution is defined as a type A curve in tympanometry examination

^b^Improvement of curve type is defined as an improvement from type B curve to type C2, C1, or A curve; or from type C2 curve to type C1 or A curve; or type C1 to A curve; or persisting type A curve at particular time point compared to the baseline

^c^Improvement of curve type is defined as an improvement from type B curve to type C2, C1, or A curve; or from type C2 curve to type C1 or A curve; or type C1 to A curve; or persisting type A curve at particular time point compared to the previous visit

#### Correlation between ear pain and other relevant symptoms and the change of MEE

**Fig. 4:**
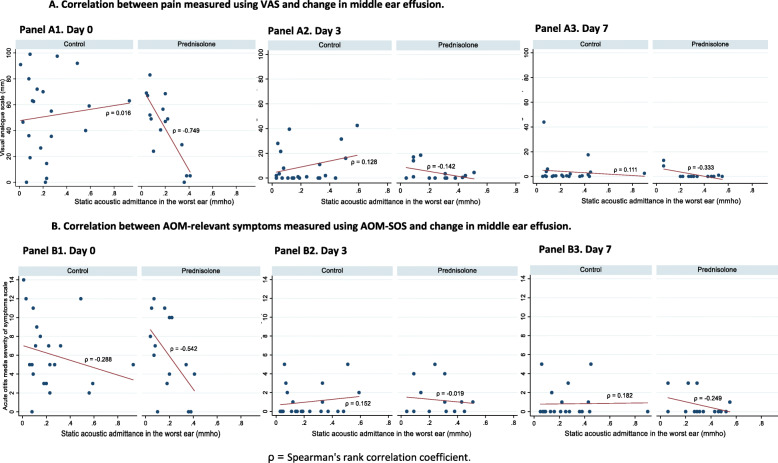
Correlation between pain or AOM-relevant symptoms and change in middle ear effusion

We expected there would be a strong negative correlation between lower VAS (no or less pain) scores or lower AOM-SOS scores (no or less AOM-relevant symptoms) and higher values of SAA (no middle ear effusion), particularly at early timepoints. However, our analysis demonstrated only a small correlation between pain and other AOM-relevant symptoms and MEE at day 3 and 7 (see Fig. 4), as well as later (day 30 and 90) (see Additional file 1).

For clinical outcomes of this pilot study, we found that prednisolone reduced pain severity at day 3 by 7 mm (MD − 7.37, 95% CI − 13.36 to − 1.39, *p* = 0.018). Although it was less than 10 mm (the minimum clinical important difference), the CI included 10 mm which indicates there may be a clinically important reduction in pain severity. There was no statistically significant difference in the proportion of children with ongoing pain at days 1, 3, 5, 7 and 14 (see Additional file 1). Neither was there a difference between groups in reduction of pain severity at these timepoints except day 3. The results remained consistent after being adjusted for the baseline pain score (ANCOVA).

### Harm

Regarding harm or adverse events (AEs), there were more children in the prednisolone group that experienced drowsiness (RR 1.77, 95% CI 1.11 to 2.81, *p* = 0.016) (Table 7). This can be translated as for every three paediatric AOM patients who received oral prednisolone, one additional patient experienced drowsiness (number needed to harm/NNTH of 3). However, there were no serious AEs of any kind attributed to study medication. There were no significant differences in the proportion of children experiencing other AEs between two groups, including those AEs commonly attributed to short-course oral corticosteroids use such as gastrointestinal problems (e.g. nausea, vomiting, diarrhoea). We found one child in the prednisolone group who had microcytic hypochromic anemia at day 6 based on low haemoglobin and serum iron counts. He was referred to a paediatrician who then confirmed this was most likely caused by iron deficiency and not by steroid intake.

**Table 7:** Adverse events in the study

Adverse events	Prednisolone group (*n* = 31)				Control (*n* = 31)			
	No pts^a^	Day 1–3	Day 4–7	Day 8–14	No pts^a^	Day 1–3	Day 4–7	Day 8–14
Increased appetite	18	11	15	17	17	9	10	7
Increased urine volume	11	8	9	7	14	12	7	4
Weight gain	13	5	6	6	11	4	4	10
Gastritis	2	3	2	0	4	2	0	0
Nausea	6	4	1	0	7	5	4	1
Vomiting	5	3	0	0	3	3	1	1
Diarrhoea	2	3	1	1	4	1	0	0
Drowsiness	23	14	9	6	13	16	10	5
Anxiety	4	3	3	3	6	3	1	1
Headache	4	1	0	1	6	7	2	3
Skin rash	3	0	1	0	0	2	0	0
Candidiasis	3	2	1	0	1	1	0	0
Dry mouth	7	5	4	0	6	6	3	1
Sleep disturbance	17	5	5	3	10	9	1	0
Others	1	0b	1	0	0	0	0	0
Serious adverse effects	0	0	0	0	0	0	0	0

^a^Total number of patients having the adverse event during the first 2 weeks

^b^Patient was detected to have anemia at day 6 (no baseline Hb count was identified) at the primary care centre and was referred to paediatrician

*Pts* patients

### Sensitivity analysis

Due to interference of the principal investigator who was not blinded to study medication allocation, we conducted a sensitivity analysis by excluding the children who received interference. This did not change the results.

## Discussion

Our pilot study met our two key objectives related to the feasibility of the full-size RCT and mechanistic effect of corticosteroids on MEE. We found that less than 40% of screened children were recruited. Most physicians and parents rated study procedures as ‘neutral’ to ‘easy’. However, most healthcare personnel found time constraints due to workload as their most common obstacle in the study. The sample size needed to power a full randomised controlled trial is lower than anticipated. Most children completed the study (97%), and a minority received concomitant oral corticosteroids and other co-treatments from an unblinded researcher. There is the potential that corticosteroids may reduce pain severity at day 3 and improve tympanometry curve by day 7. We found only a small correlation between the change in MEE and pain and other AOM-relevant symptoms. We also found drowsiness as the most common side effect of oral corticosteroid.

There were several limitations of this pilot study. Our recruitment rate was low and similar (38.5%) to other studies conducted in developing countries [39]. This could be because we started recruiting at hospital centres, but well-implemented national coverage insurance required patients to access healthcare services via primary care centres. It could also be due to (1) low research awareness/interest among physicians, nurses and patients [40]; (2) workload of physicians and nurses; (3) cultural factors (e.g. patients seeking family/relatives’ consent to participate in research); and (4) insufficient clinical trial facilities [40]. To improve recruitment, future studies could (1) recruit from more primary care centres; (2) provide incentives for participating healthcare personnel despite insufficient evidence of effects on recruitment rate [41]; and (3) simplify the study process (simplifying and improving the layout of CRFs and symptom diary and allocating research assistants to support study procedures including randomisation).

We could not provide a matched placebo control. Parents’ subjective responses to pain assessment could have been biased by this, as they knew which treatment their child received. However, we plan to use a placebo in the full RCT where we will involve an independent specialised drug manufacturer to provide a placebo that is similar in form and taste with oral prednisolone.

Four children received additional oral corticosteroids. Although we asked parents to contact us before they sought consultation from other physicians for any relevant or other concurrent conditions, this was not sufficiently implemented. Therefore, for the full RCT, we will provide a handy information card that provides detailed information about the study, including medication that should not be given. This card should be shown to any physicians that the parent consults.

Imbalanced randomisation meant that there were more children in the control group who had low parental education level, were exposed to parental smoking and received amoxicillin and acetaminophen compared to those in the prednisolone group. One potential contributing factor was that, by chance, most children in the control group were recruited from the primary care centres. Out of 31 children in the control group, 13 were recruited from the primary care centres (55%) and out of 31 children in the prednisolone group, only six were recruited from primary care centres (19%). Low parental education level may be associated with higher rates of passive smoking in the control group. This was supported by evidence showing education attainment and length of education negatively correlated with smoking behaviour [42]. Parental smoking has been identified as a strong risk factor for AOM [43–45]. First line antibiotics for AOM and analgesics in primary care centres in Indonesia are amoxicillin and acetaminophen [15]. This is consistent with the antibiotic recommended for AOM in guidelines, but at a lower dose (50 mg/kg body weight/day) [15]. This chance imbalance in randomisation will likely be reduced by stratification on the type of healthcare facility (primary versus secondary/tertiary care centre).

The last limitation was missing tympanometry values, which was caused by the inability of tympanometry to detect key values (i.e. SAA, middle ear pressure) in some severe cases of MEE. However, this problem was detected after several incomplete test values which resulted in missing data from several cases. Despite this, given the lack of clinical benefit of this examination in AOM cases, the need of specific skills and facilities, and cost, we do not intend to include the tympanometry measurement in the full RCT.

We also found positive impacts of this study. The first strength of this study is its ability to identify potential issues particularly in the recruitment and data collection which allows us to modify study procedures and strategies for a successful implementation of the full RCT [46]. Secondly, we also believe this pilot study has introduced a clinical trial to healthcare personnel at several levels of healthcare service in Indonesia. We expect this will trigger their interest in research since they now have some knowledge and experience in conducting a clinical trial.

Our original primary outcome was the proportion of children with ongoing pain that has not reduced by the minimum clinically important amount (VAS score of 10 mm) by day 3. However, our pilot study demonstrated that the majority of the children had their pain significantly improved at day 3. Therefore, we will change the primary outcome in the main RCT to be the proportion of children with persisting pain (defined as the VAS score greater than 5 mm). We will retain the secondary outcome, that is the reduction of pain intensity using VAS, which will allow us to identify the effectiveness of oral prednisolone to improve pain by the previously-defined minimum clinically important amount.

As we included different levels of healthcare service in several districts in Jakarta in the pilot study, we are confident that it is feasible to conduct the full RCT in other cities in Indonesia, particularly on Java. Training prior to the trial customised to education level of healthcare personnel is crucial. The availability of an otoscope will be a potential limitation for a large-scale RCT.

This study also showed that the incidence of severe AOM (47/62; 76%) was higher compared to other trials referenced in this study that were mainly conducted in developed countries. If a large RCT is conducted in Indonesia or other developing countries with similar AOM characteristics, then it will predominantly evaluate the effects of oral corticosteroids as an addition to antibiotics.

Decongestants and/or antihistamines were commonly prescribed at baseline consultation. Most AOM patients experienced symptoms of the common cold which could explain this finding. Evidence shows that the combination of decongestants and antihistamine is beneficial for general recovery in adults and older children with common cold, but not in young children (age < 5 years) [47]. Regarding its effects for AOM, decongestants and/or antihistamines have only a modest benefit in reducing the risk of persistent AOM in 2 weeks with significant adverse events overweighting the benefits [19].

We re-introduced the use of pain assessment tools (i.e., VAS and AOM-SOS) which have not been routinely used in the management of children with AOM in our clinical setting. Most parents assessed their children’s pain by observation. Only few older children (5/62; 6%) did a self-rated pain assessment.

We also found that most parents and physicians preferred to use a numeric or facial pain scale. One of the commonly used self-report numeric scales for acute pain is the 11-point Numeric Rating Scale (NRS-11). Children, particularly aged 6 years and older, determine their pain intensity by choosing a number between 0 (representing ‘no hurt’) and 10 (representing ‘the worst hurt’) scale [48, 49]. For facial pain scale, a Faces Pain Scale-Revised (FPS-R) was recommended as a self-report acute pain scale for children aged 7 years and older. It consists of six faces ranging from ‘no pain’ to ‘very much pain’, where each face was represented by numbers of 0-2-4-6-8-10 [48, 50]. However, since there was no strong evidence supporting the recommendation of any particular parent-report pain assessment for paediatric population with acute pain, we still consider it appropriate to use VAS and AOM-SOS for the full RCT. This pilot study also verified that these pain assessment tools were successfully implemented by the parents in assessing pain.

We did not find clinically significant benefits of tympanometry examination for the management of AOM. It was costly and difficult to implement in children experiencing pain. Evidence demonstrates that only certain children may be at risk of prolonged MEE resolution (children with AOM aged < 2 years or, children with recurrent AOM). Therefore, tympanometry examination should be prioritized for those children and not to be generalised for all AOM cases [9, 51]. We will further investigate any prognostic factors and characteristics of our study participants in the tympanometry sub-study that may influence the improvement or prolongation of MEE.

We found drowsiness as the most common side effect of oral corticosteroid, which is consistent with AEs related to a short-term use of oral corticosteroids in other studies [21, 26]. However, this requires further investigation since more children in the prednisolone group received decongestants and/or antihistamines, and drowsiness has been identified as common side effect of decongestants and/or antihistamines with risk up to eight-fold of risk in the treatment group [19, 52, 53].

Compared to other feasibility interventional studies, our pilot study had similar recruitment issues which required additional recruitment time and modification of the recruitment strategy [54]. Our study had a lower recruitment rate compared to other feasibility studies [55]. However, like other studies, our pilot study had a low rate of incomplete outcome data (6%) [54].

## Conclusions

Our pilot study shows that it is feasible to conduct a large, pragmatic, randomised, double-blind placebo-controlled trial. However, several modifications should be made to improve feasibility: simplifying study procedures, improving the layout of CRF and symptom diary, recruiting through primary care centres, stratifying children based on severity and healthcare centre level and the use of placebo as a control. We will use VAS and AOM-SOS as pain assessment tools since there is no strong evidence recommending a particular parent-report assessment tool for children with acute pain. The sample size required is less than we anticipated due to the high proportion of severe AOM cases, and it is not necessary to use tympanometry for a future trial. Based on the findings and results from this pilot study, we modified our protocol for the full RCT (see Additional file 2).

Our clinical results do not rule out the benefit of oral corticosteroids for AOM. However, the signal of its benefits is small. We also identified drowsiness as one side effect of a short-term us of oral corticosteroids, with no excess of other AEs commonly attributed to short-course oral corticosteroids use (i.e. nausea, vomiting, diarrhoea) found. Therefore, our pilot study confirms the importance of conducting our planned full RCT to assess the actual effects, both benefits and harm, of oral corticosteroids for children with AOM.

## Supplementary information

**Additional file 1.** Clinical outcomes of the pilot OPAL study

**Additional file 2.** Protocol of the main OPAL study

## Data Availability

Our protocol is published in BMC Pilot and Feasibility Studies [27] and also can be accessed at https://research.bond.edu.au/en/publications/oral-prednisolone-for-acute-otitis-media-in-children-opal-study-a. The datasets generated and/or analysed during the current study are not publicly available because this is a pilot study which the clinical results could be misinterpreted. However, they are available from the corresponding author on reasonable request.

## Abbreviations

AE: Adverse event
AOM: Acute otitis media
AOM-SOS: Acute Otitis Media Severity of Symptoms scale
ARI: Acute respiratory infection
BUHREC: Bond University’s Human Research Ethics Committee
CI: Confidence interval
CRF: Case report form
ENT: Ear, nose, and throat
FPS-R: Faces Pain Scale-Revised
GP: General practitioner
HIV: Human immunodeficiency virus
MD: Mean difference
MEE: Middle ear effusion
Pts: Patients
NRS-11: 11-Point Numeric Rating Scale (NRS-11)
P: *P* value
RR: Relative risk
RCT: Randomised controlled trial
SAA: Static acoustic admittance
VAS: Visual analogue scale
