# Supplementary material for: Oral prednisolone for acute otitis media in children: a pilot, pragmatic, randomised, open-label, controlled study (OPAL study)
Source: Pilot Feasibility Stud. 2020 Aug 29;6:121. doi: 10.1186/s40814-020-00671-5 (PMC7455987; doi:10.1186/s40814-020-00671-5)
Supplement: Supplementary file 1 — Additional file 1. Clinical outcomes of the pilot OPAL study [file 40814_2020_671_MOESM1_ESM.pdf]

Appendix 1. Clinical outcomes of the pilot OPAL study.

Figure 1 Panel A. Correlation between pain measured using VAS and change in middle ear effusion*

A1. Visit 0 (Day 0)

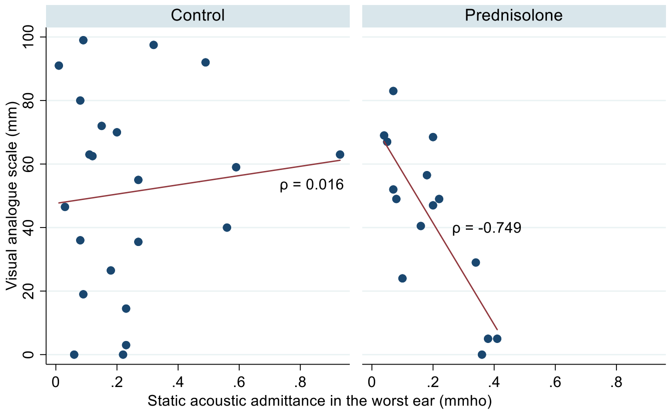

A2. Visit 1 (Day 3)

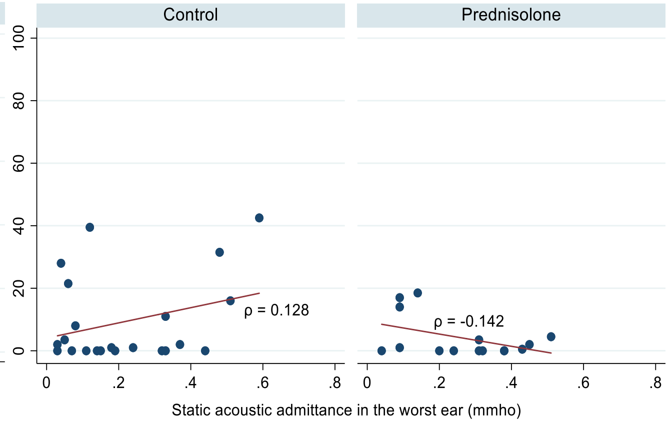

A3. Visit 2 (Day 7)

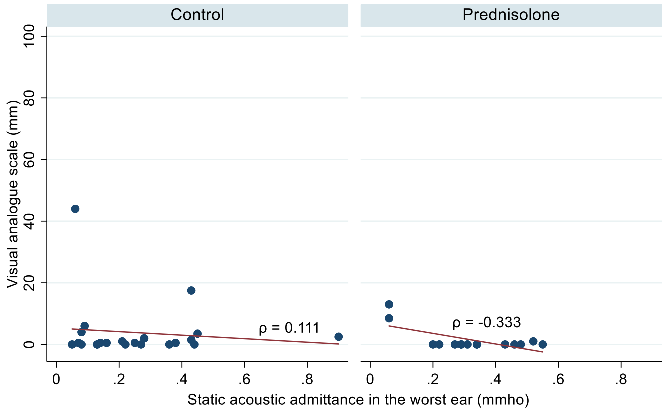

A4. Visit 3 (Day 30)

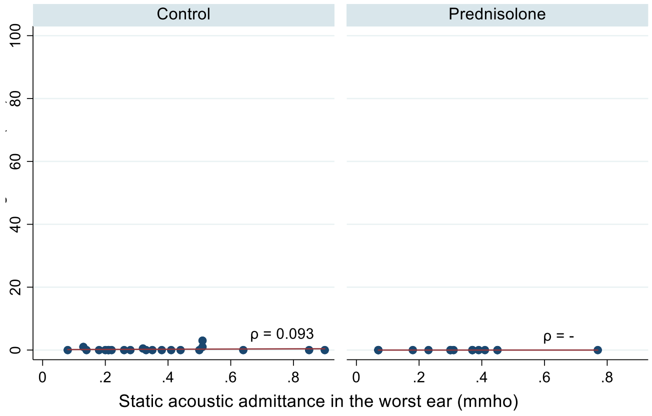

A5. Visit 4 (Day 90)

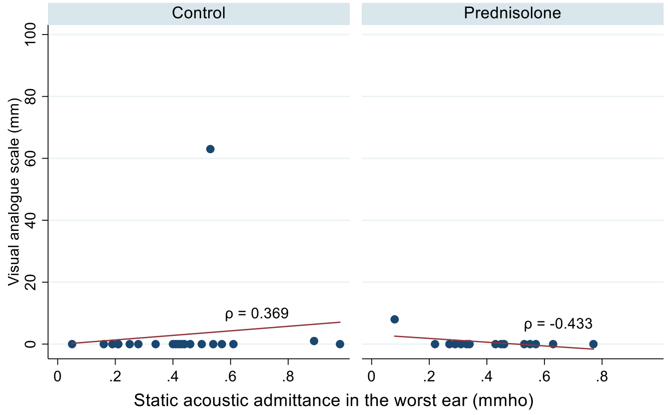

ρ = Spearman's rank correlation coefficient

Figure 1 Panel B. Correlation between AOM-relevant symptoms measured using AOM-SOS and change in middle ear effusion.

B1. Visit 0 (Day 0)

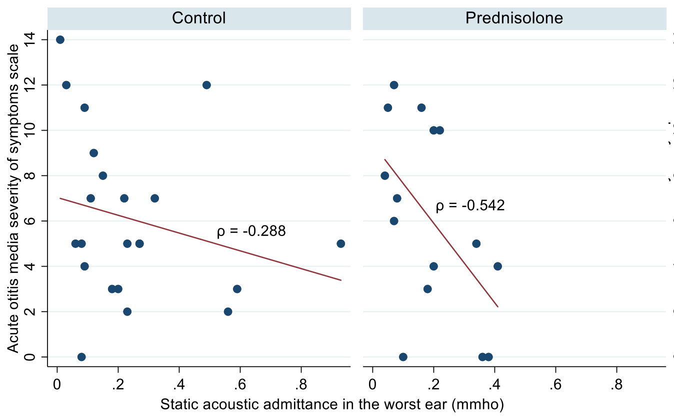

B2. Visit 1 (Day 3)

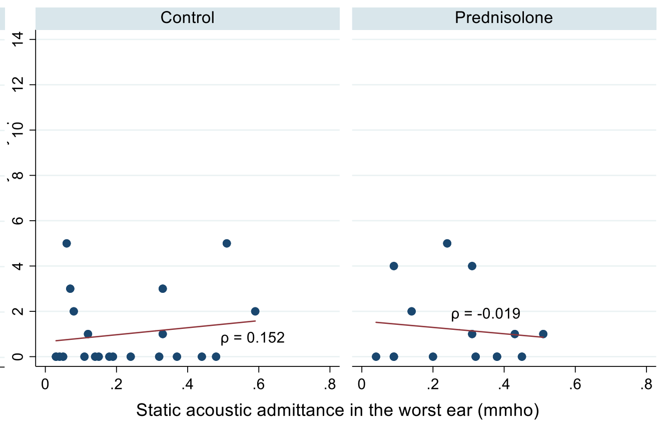

B3. Visit 2 (Day 7)

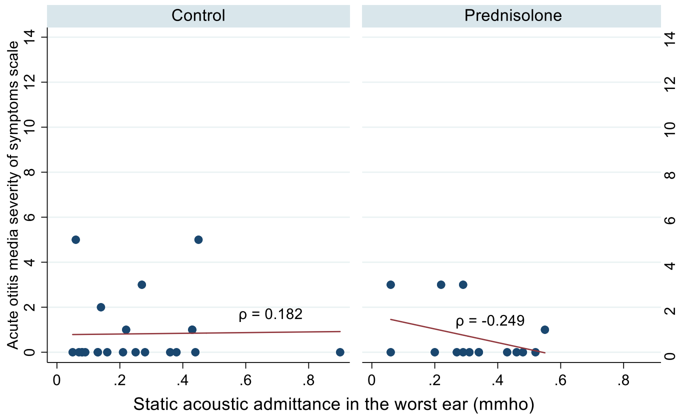

B4. Visit 3 (Day 30)

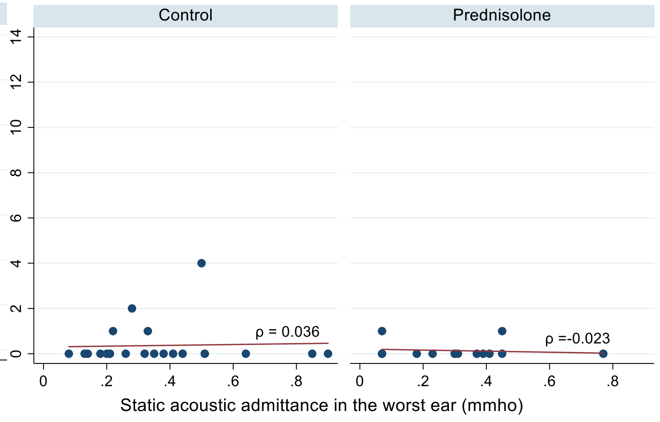

B5. Visit 4 (Day 90)

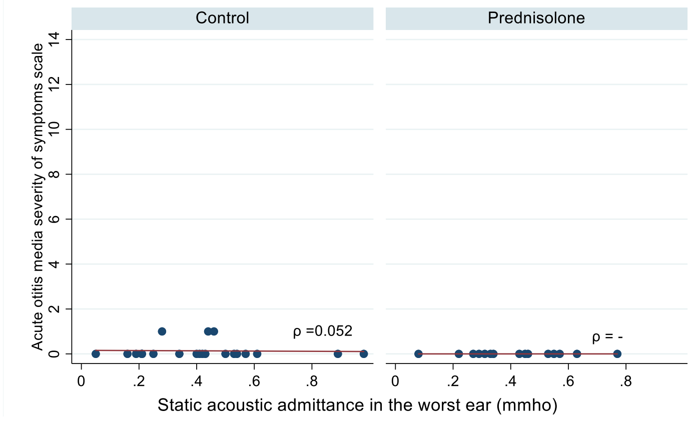

ρ = Spearman's rank correlation coefficient

Table 1 Panel A – Clinical binary outcomes

Clinical outcomes*	Prednisolone (n=29)	Control (n=31)	Effect estimate (relative risk)	p value
**Proportion of children with pain that has not reduced by 10 mm VAS at Day 3 (n; %)**	3 (10)	5 (16)	1.07 (0.88, 1.30)	0.71
**Proportion of children with pain represented by VAS ≥ 5mm (n: %)**				
Day 0 (Visit 0)	27 (93)	27 (87)	1.07 (0.90, 1.26)	
Day 1	24 (83)	22 (71)	1.17 (0.88, 1.54)	
Day 3 (Visit 1)	6 (21)	14 (45)	0.46 (0.20, 1.03)	0.06
Day 5	3 (10)	8 (28)	0.37 (0.11, 1.27)	
Day 7 (Visit 2)	2 (7)	5 (17)	0.40 (0.08, 1.89)	
Day 14	1 (3)	2 (7)	0.50 (0.05, 5.21)	
**Initiation of antibiotics or second-line antibiotics during Visit 1 to Visit 2 (n; %)**	9 (31)	10 (32)	0.96 (0.46, 2.02)	> 0.999
Initiation of antibiotic for mild AOM in Visit 1 or Visit 2 (n; %)**	2 (33)	6 (86)	0.39 (0.12, 1.25)	0.11
**Complications (n; %)**	1 (3)†	0 (0)		0.49
**Recurrence (n; %)**				
Day 30 (Visit 3)	1 (3)	2 (7)	0.50 (0.05, 5.21)	> 0.999
Day 90 (Visit 4)	5 (17)	2 (7)	2.50 (0.53, 11.86)	0.42
**Other descriptive outcomes**				
**Improvement of otoscopic findings compared to previous visit (n; %)**				
Day 3 (Visit 1)	23 (79)	17 (55)		
Day 7 (Visit 2)	15 (52)	15 (52)		
Day 30 (Visit 3)	22 (76)	22 (76)		
Day 90 (Visit 4)	22 (76)	24 (83)		
**Improvement of otoscopic findings compared to baseline (n; %)**				
Day 3 (Visit 1)	23 (79)	17 (55)		
Day 7 (Visit 2)	21 (72)	21 (72)		
Day 30 (Visit 3)	24 (83)	25 (86)		
Day 90 (Visit 4)	26 (90)	27 (93)		
**Tympanic membrane perforation (n; %)**				
Day 0 (Visit 0)	11 (38)	8 (26)		
Day 3 (Visit 1)	4 (14)	6 (19)		
Day 7 (Visit 2)	3 (10)	3 (10)		
Day 30 (Visit 3)	1 (3)	2 (7)		
Day 90 (Visit 4)	1 (3)	1 (3)		
**Treatment given by doctors in the study in two weeks following initial visit (n; %)**				
Acetaminophen	9 (31)	17 (55)		
NSAIDs	5 (17)	8 (26)		

Decongestants and/or antihistamine	27 (93)	29 (93)
Cough medicine	24 (83)	21 (68)
Antibiotic ear drops	8 (28)	8 (26)
Nasal topical decongestant	6 (21)	5 (16)
Nasal topical corticosteroid	8 (28)	3 (10)
Additional oral prednisolone	0 (0)	1 (3)
Vitamins or herbals	4 (14)	10 (32)
Ear diathermy	0 (0)	2 (6)
Inhalation	1 (3)	1 (3)
Others‡	4 (14)	7 (23)
**Treatment given by other doctors or self-medication/over the counter in two weeks following initial visit (n; %)**		
Antibiotics	1 (3)	1 (3)
Acetaminophen	2 (7)	1 (3)
NSAIDs	0 (0)	0 (0)
Decongestants and/or antihistamine	4 (14)	2 (6)
Cough medicine	5 (17)	4 (13)
Antibiotic ear drops	0 (0)	1 (3)
Nasal topical decongestant	0 (0)	0 (0)
Nasal topical corticosteroid	0 (0)	0 (0)
Additional oral prednisolone	2 (7)	0 (0)
Vitamins or herbals	3 (10)	2 (6)
Ear diathermy	0 (0)	0 (0)
Inhalation	0 (0)	0 (0)
Others‡	2 (7)	0 (0)
**Additional visit required (n; %)**	6 (21)	10 (32)

*Two missing data from control group after Day 3 made total number of control group 29; **There were 6 cases of mild AOM in the prednisolone group and 7 cases in the control group; †Tympanic membrane perforation at Day 30, which healed at Day 90; ‡Others (e.g. mefenamic acid, nasal wash).

Table 1 Panel B – Clinical continuous outcomes

Clinical outcome*	Prednisolone (n=29)	Control (n=31)	Unadjusted mean differences	p value	Adjusted mean difference**	p value
**Pain measured by VAS (mm) mean (SD)†**						
Day 0 (Visit 0)	48.9 ± 27.1	49.6 ± 31.1	-0.63 (-15.74, 14.48)	0.93		
Day 1	25.7 ± 20.7	23.5 ± 20.9	2.14 (-8.63, 12.91)	0.69	2.33 (-7.54, 12.19)	
Day 3 (Visit 1)	3.9 ± 5.6	11.3 ± 15.2	-7.37 (-13.36, -1.39)	0.017	-7.36 (-13.40, -1.33)	0.018
Day 5	4.2 ± 15.2	5.5 ± 12.7	-1.27 (-8.63, 6.08)	0.73	-1.20 (-8.38, 5.98)	
Day 7 (Visit 2)	1.1 ± 2.8	3.9 ± 9.3	-2.84 (-6.47, 0.78)	0.12	-2.84 (-6.51, 0.82)	
Day 14†	0.6 ± 2.8	0.8 ± 1.9	-0.21 (-1.45, 1.04)	0.74	-0.21 (-1.46, 1.05)	
Day 30 (Visit 3)	1.5 ± 6.8	0.2 ± 0.6	1.33 (-1.21, 3.87)	0.30	1.32 (-1.24, 3.89)	
Day 90 (Visit 4)	2.3 ± 10.3	2.2 ± 11.7	0.05 (-5.76, 5.86)	0.99	0.04 (-5.81, 5.89)	
**Symptoms measured by AOM-SOS mean (SD)†**						
Day 0 (Visit 0)	6.4 ± 3.8	6.1 ± 3.6	0.32 (-1.61, 2.24)	0.74		
Day 1	3.2 ± 3.0	3.1 ± 2.5	0.07 (-1.35, 1.50,)	0.92	-0.03 (-1.31, 1.25)	
Day 3 (Visit 1)	1.2 ± 1.9	0.9 ± 1.6	0.27 (-0.63, 1.17)	0.55	0.22 (-0.64, 1.09)	0.60
Day 5	1.1 ± 1.7	0.7 ± 1.2	0.38 (-0.39, 1.15)	0.33	0.38 (-0.40, 1.16)	
Day 7 (Visit 2)	0.5 ± 0.9	0.8 ± 1.5	-0.31 (-0.96, 0.34)	0.34	-0.31 (-0.97, 0.35)	
Day 14†	0.9 ± 2.3	0.2 ± 0.5	0.72 (-0.16, 1.61)	0.11	0.71 (-0.16, 1.57)	
Day 30 (Visit 3)	0.2 ± 0.6	0.5 ± 1.2	-0.27 (-0.79, 0.24)	0.29	1.34 (-1.22, 3.90)	
Day 90 (Visit 4)	0.1 ± 0.4	0.1 ± 0.3	0.03 (-0.17, 0.23)	0.73	0.11 (-5.70, 5.92)	
**Time to pain resolution‡ (median days)**	2	3	-1	0.71		

*Two missing data from control group after Day 3 made total number of control group 29; **Adjusting for the baseline and intervention allocation; †VAS (ranged 0 to 100 mm), higher score representing worse pain. AOM-SOS (ranged 0 to 14 points), higher score for worse symptom; ‡We analysed 55 out of 60 children (28 prednisolone and 27 control): 5 children not included were four controls (two left the study and two did not have pain resolution in two weeks) and one prednisolone did not have pain resolution in two weeks observation.
