# Supplementary material for: Oral prednisolone for acute otitis media in children: a pilot, pragmatic, randomised, open-label, controlled study (OPAL study)
Source: Pilot Feasibility Stud. 2020 Aug 29;6:121. doi: 10.1186/s40814-020-00671-5 (PMC7455987; doi:10.1186/s40814-020-00671-5)
Supplement: Supplementary file 2 — Additional file 2. Protocol of the main OPAL study [file 40814_2020_671_MOESM2_ESM.pdf]

Additional file 2. Protocol of the main OPAL study.

Oral prednisolone for acute otitis media in children: a proposed pragmatic, parallel, randomised, double-blind, placebo-controlled study (OPAL study)

Ranakusuma RW[1,2], McCullough AR[1], Safitri ED[2], Pitoyo Y[2], Widyaningsih W[2], Del Mar CB[1], Beller EM[1].

[1]Institute for Evidence-Based Healthcare, Bond University, Robina, Queensland, Australia.

[2]Clinical Epidemiology and Evidence-Based Medicine Unit, Dr Cipto Mangunkusumo Hospital – Faculty of Medicine Universitas Indonesia, Jakarta, Indonesia.

1. Executive Summary

Background: Acute otitis media (AOM) is an acute middle ear infection particularly found in children. Antibiotics are commonly prescribed, despite most cases being sufficiently managed by observation and adequate pain management. Due to weak effects and potential risks of antibiotic use, such as side effects and antibiotic resistance, an alternative non-antibiotic treatment for AOM is required. Corticosteroids could be a candidate for AOM, yet there is insufficient evidence on their efficacy. We have planned a high-quality clinical trial to test the effectiveness of oral corticosteroids in improving clinical outcomes including reducing middle ear effusion (MEE) in children with AOM. Our trial is justified by our pilot study showing that oral corticosteroids potentially reduce pain intensity at Day 3 and improve tympanometry results at Day 7, although the observed difference was small. This adequately powered clinical trial will allow us to definitively conclude whether corticosteroids are an effective treatment for AOM along with its effect size.

Methods: This multicentre, pragmatic, randomised, double-blind, placebo-controlled study will recruit 444 children aged 6 months to 12 years with onset of AOM within 48 hours. We will stratify children based on healthcare facility type and AOM severity. Children will be randomly allocated to receive either prednisolone or placebo plus observation in mild AOM and either prednisolone or placebo plus antibiotics in severe AOM. Our primary aim is to assess the effectiveness of oral corticosteroids in improving pain at three days after randomisation. Secondary aims are to assess: (1) the effectiveness of corticosteroids in improving pain at other time points, reducing pain intensity and overall AOM-relevant symptoms, reducing time to pain resolution, and the need for antibiotic initiation (mild AOM) or second-line antibiotics (severe AOM), AOM complications and recurrence; and (2) the adverse effects of corticosteroids.

Discussion: High rates of antibiotic use for AOM indicates the need for alternative treatments. Due to the inflammatory nature of AOM, corticosteroids may be a suitable treatment. If our study shows positive results with enough benefits and sufficient effect size outweighing harm, we can offer oral

corticosteroids as one potential alternative treatment for AOM and thereby reduce antibiotic use. This study will contribute to adding to the range of AOM non-antibiotic treatment and updating current AOM management guidelines which do not make recommendations about oral corticosteroids for AOM.

Keywords: Otitis media, Acute disease, Glucocorticoids, Anti-bacterial agents, Clinical trial protocol.
Trial registration: Not yet.

2. Background

Acute otitis media (AOM) is an acute middle ear infection commonly found in children [1-3]. High rates of antibiotic prescribing are evident [4,5], although only one third of children with AOM, namely those who have severe cases, will likely benefit from antibiotics [4,6,7].

Due to the potential harm of antibiotic use and its weak benefits in improving clinical outcomes, alternative treatments for AOM, such as corticosteroids as anti-inflammatory agents, are needed. Due to the uncertain effects of corticosteroids for AOM [8,9], we plan to conduct a high-quality clinical trial to assess the effectiveness of corticosteroids for children with AOM. We recently tested the feasibility of this in a pilot, pragmatic, randomised, single-blind, controlled trial, which demonstrated it was feasible to conduct all planned procedures and measurement for a high-quality clinical trial. We found that oral prednisolone may potentially reduce pain intensity at day 3. We also found drowsiness as the most common adverse event in children in the prednisolone group, with no other adverse events commonly attributed to short-course oral corticosteroids nor serious adverse effects found. Therefore, we will conduct a large, pragmatic, double-blind, randomised, placebo-controlled to test the effectiveness of corticosteroids, including benefits and harm, in children with AOM.

3. Methods/Design

3.1 Aim and objectives

We aim to assess the effectiveness of oral corticosteroid as a monotherapy for children with mild AOM and as an addition to antibiotics for severe AOM.

Our primary objective is to assess the effectiveness of corticosteroids in improving pain at three days after randomisation.

Our secondary objectives are to assess: (1) the effectiveness of corticosteroids in: (a) improving pain at other time points; (b) reducing pain intensity; (b) reducing overall AOM-relevant symptoms; (c)

reducing duration to pain resolution; (d) reducing the need of antibiotic initiation for children with mild AOM or second-line antibiotics treatment for children with severe AOM after 48-hour observation; (e) reducing the risk of AOM complications; and (f) reducing the risk of AOM recurrence; and (2) the adverse effects of corticosteroids.

3.2 Design

We will conduct a large, parallel, pragmatic, multicentre, stratified, double-blind, randomised, placebo-controlled with an allocation ratio of 1:1, where children will be randomly allocated to either receive oral prednisolone or placebo.

3.3 Study setting

This multicentre study will be conducted at six primary care centres and two hospitals in Central and East Jakarta, Indonesia. We will include four healthcare centres which participated in our previous pilot study (two primary care centres and two hospitals). We will identify four other primary care centres based on an adequate number of paediatric AOM patient visits and the convenience of study monitoring.

We will include general practitioners (GPs) from the primary care centres and ear-nose-throat (ENT) specialists from the hospitals in the recruitment and assessment process of the study. We will also include nurses for study randomisation and pharmacists for study medication storage, preparation, and dispensing.

3.4 Eligibility criteria

3.4.1 Inclusion criteria

We will include children aged 6 months to 12 years old with AOM, defined as current onset (within 48 hours) of AOM-relevant symptoms (e.g. earache, ear discharge, ear tugging/rubbing or irritability in non-verbal children). Otoscopic findings of acute inflammation (e.g. erythema) and middle ear effusion (e.g. bulged tympanic membrane, air-fluid level) will confirm the diagnosis. Due to potential obstacles in otoscopic assessment in children (e.g. uncooperative, narrow ear canal, ear wax) and the pragmatic nature of this study in reflecting real practice in the management of AOM, we will include children with symptoms strongly indicating AOM whose diagnosis cannot be confirmed using an otoscope, and diagnose these children with suspected AOM.

3.4.2 Exclusion criteria

We will exclude children who (1) have major and severe medical conditions (e.g. heart diseases, kidney failure, tuberculosis), (2) are immunocompromised (e.g. HIV infection, under cancer treatment), (3) have congenital malformations and/or syndromes (e.g. cleft palate, Down syndrome), (4) have high risk of strongyloidiasis infections, (5) have ear ventilation tube(s), (6) have been exposed to persons with varicella (chicken pox) or active Zoster infection in the past three weeks without prior varicella immunisation or infection, (7) have taken systemic (oral, injection) or topical steroids in the preceding 4 weeks, (8) have taken antibiotics in the preceding 2 weeks, and (9) are hypersensitive to prednisolone or prednisone, or other corticosteroids.

3.5 Interventions

3.5.1 Prednisolone

Prednisolone tablets (Lupred®5) will be given as a single daily dose of 1–2 mg/kg of body weight for 5 days. A wide therapeutic dose window of prednisolone allows the simplification of randomisation and dispensing as follows: 10 mg/day for children aged 6 months to up to 2 years; 20 mg/day for children aged 2up to 6 years; and 30 mg/day for children aged 6 to 12 years [10]. We strongly recommend children take prednisolone as a whole (single dose) in the morning (6 to 8 am) to minimise the risk of Hypothalamic–Pituitary–Adrenal (HPA) axis suppression and for the convenience of both the patients and their parents or care givers. We will provide a liquid sweetener to be added to the study medication to make it more palatable for the children. The parents or care givers will be asked to give the prednisolone with food or milk to decrease the risk of gastrointestinal disturbance.

Children with mild AOM who are randomly allocated to the intervention arm will receive prednisolone plus expectant observation, whilst those with severe AOM will receive prednisolone plus antibiotics.

3.5.2 Placebo

Children in the control group will receive a 5-day course of matched placebo which has similar form, colour, and taste to prednisolone.

Children with mild AOM who are randomly allocated to the control arm will receive placebo plus expectant observation, whilst those with severe AOM will receive placebo plus antibiotics.

3.6 Criteria for study drug discontinuation or modification

If a child vomits less than 30 minutes after taking a dose of study medication, parents will be instructed to give another dose and report to the research team for an additional dose for completing a 5-day course of study medication. However, if a child vomits after 30 minutes, parents should not give another dose until the next dose on the next morning. If a child keeps vomiting or experiencing other unfavourable effects (e.g. nausea, diarrhea) after receiving the study medication, parents should contact the research team. If the parents forget to give study medication, they can give the missed dose as soon as they remember on the same day. Any modification of taking study medication, such as above, should be recorded in the symptom diary.

If there are any adverse events and adverse drug reactions where the research team assesses discontinuation of study medication is required, the treatment will be discontinued; however, follow-up will continue, where possible.

3.7 Strategies to improve adherence to the intervention protocol

We will remind parents to complete a symptom diary and give the study medication to their children every morning for 5 days by sending them text-message reminders every morning for 2 weeks. We have also added a note at the bottom of the questionnaire in the first booklet of the symptom diary to remind them to give the study medication every morning for 5 days. We will ensure that all parents have enough study medication to complete a 5-day course of intervention.

We will ask parents to keep the paper wrap packaging from the 5-day course of study medication. We will collect these paper wraps during their second visit at day 7 to check the adherence of the study participants in taking study medication per protocol.

We will provide an information card for each patient at the baseline visit. This card will provide a summary of the study, including the intervention used in the study, and will state no additional oral corticosteroid should be prescribed during the study where possible. The parents should take and show the information card to any doctor consultations, to avoid additional oral corticosteroid intake. The parents should record all prescribed medications from these consultations or any over-the-counter medications in the symptom diary.

3.8 Concurrent treatment

Physicians may prescribe medications for symptoms (e.g. antipyretic, analgesic, decongestant, inhalation) according to their usual practice and record all medication prescribed in the outcome form, which is one of the case report forms (CRFs) developed for the study.

The physicians must not prescribe oral corticosteroids. The nurses and research assistant will cross-check all prescriptions before dispensing them to the parents, to ensure no oral corticosteroids are prescribed. If a child unintentionally receives any additional oral corticosteroids, we will not exclude the child from the study due to intention-to-treat analysis of the study. At the end of the study, we will identify any additional oral corticosteroids prescribed either from participating physicians or other physicians using the outcome form and symptom diary. All decisions on prescribing by physicians will be made prior to randomisation.

3.9 Outcomes

3.9.1 Primary outcome

For the primary objective of assessing the effectiveness of corticosteroids in improving pain, we will assess the proportion of children with ongoing pain represented by visual analogue scales (VAS) score of 5 mm or more [11] at day 3 following the randomisation. We will assess this outcome using the VAS from the symptom diary.

3.9.2 Secondary outcomes

For the secondary objective of assessing the effectiveness of oral corticosteroids in improving pain at other time points, reducing pain intensity, overall AOM-relevant symptoms, and time to pain resolution, we will measure: (1) the proportion of children with ongoing pain (VAS score of ≥ 5 mm) using VAS in the symptom diary at 24 hours, day 5, day 7 and day 14; (2) reduction of pain intensity using VAS in the symptom diary; (3) reduction of overall AOM-relevant symptoms using the overall score of acute otitis media severity of symptoms scale (AOM-SOS); and (4) time duration to pain resolution using VAS in the symptom diary. The reduction of pain intensity and overall AOM-relevant symptom outcomes will be measured at 24 hours, day 3, day 5, day 7 and day 14. The time duration to pain resolution outcome will be measured during a 2-week observation period.

For the objective of reducing the need for antibiotic initiation for AOM, we will measure the proportion of children who require antibiotic initiation after 48-hour observation in children with mild

AOM or second-line antibiotic treatment for children with severe AOM by 2 weeks after randomisation (Day 14).

For the objective of reducing the risk of AOM complications, we will measure the proportion of children who experience any complications of AOM, such as perforation of tympanic membrane or acute mastoiditis within two weeks following the baseline visit.

For the objective of reducing the risk of AOM recurrence, we will measure the proportion of children who experience at least one new episode of AOM within 1 month and 3 months following the baseline visit.

For the objective of assessing the adverse effects of corticosteroids, we will measure the proportion of children who experience unfavourable side effects after taking the medication prescribed for the study within 2 weeks following the baseline visit.

3.10 Participant timeline

Table 1 illustrates the timeline for visits and follow-ups. Patients should visit the primary care centres or hospitals at day 3 and day 7 for re-assessment where physicians will identify the resolution of AOM symptoms (e.g. ear pain and other AOM-related symptoms) and clinical signs using an otoscope. During these visits, we will collect the first two booklets of the symptom diary. The first booklet records the symptoms, complications, side effects, other doctor consultations, and list of medications taken from baseline until day 3, whilst the second booklet records similar outcomes from day 4 to day 7. At day 3, the nurse will check the left-over study medication and ensure the study medication is available for a 5-day course of intervention. At day 7, the nurses will collect and count the paper wraps and any left-over study medication. The third booklet of the symptom diary also records similar outcomes from day 8 to day 14 and will be collected during the home visit at day 14. During the home visit, we will not provide any prescriptions. Research assistants will contact all parents at day 30 and day 90 by phone to obtain information of AOM symptoms and recurrence of AOM.

Table 1. Follow-up timeline

	Study period						
	**Enrolment Allocation**	**Post-allocation**					**Close-out**
**Timepoints**	**0 (Day 0)**	**t1 (Day 3)**	**Intervention Ends* (Day 5)**	**t2 (Day 7)**	**Home visit (Day 14)**	**t3† (Day 30)**	**t4† (Day 90)**
**ENROLMENT:**							
Eligibility screen	X						
Informed consent	X						
Allocation	X						
**INTERVENTIONS:**							
[Intervention A] Prednisolone	—	—	—				
[Intervention B] Placebo	—	—	—				
**ASSESSMENTS:**							
General examination	X	X		X			
Pain severity using VAS	X	X	X	X	X		
Overall AOM-relevant symptoms using AOM-SOS	X	X	X	X	X		
Otoscopic examination	X	X		X			
Requirement of 1st line or 2nd line antibiotic initiation		X	X	X	X		
Complications	X	X	X	X	X		
AOM recurrence						X	X
Adverse events		X	X	X	X		

*Data will be collected from the symptom diary; †The follow-up on these time-points will be conducted through phone-calls.

3.11 Sample size

Based on our initial size calculation, we needed to enrol 760 children with AOM [12]. We had assumed that 30% of participants would have severe AOM [7], however our pilot study demonstrated that 78% of our total sample was in the severe group with the risk of ongoing pain (≥ 5 mm VAS) in the control group at day 3 of 42%. Of the children with mild AOM, 57% in the control group had ongoing pain at day 3. The average proportion of children in the mild and severe groups with ongoing pain at day 3 was 45.2%. With our original assumption of 0.70 risk ratio with steroids [7], we will only need to study

201 experimental and 201 control subjects to be able to reject the null hypothesis with probability (power) 0.8 and type I error probability of 0.05. We will use an uncorrected chi-squared statistic to evaluate the null hypothesis of no difference between groups. The total sample size becomes 444 with a 10% allowance for dropouts.

The significant difference in the sample size estimation between our original sample size and the pilot study, which was conducted in an urban setting in a developing country, is mostly influenced by our original calculation that used effect assumptions originating from a meta-analysis of clinical trials conducted in developed countries. This indicates that the study sample size may change depending on the settings where the study will be conducted. Table 2 presents our sample size assumptions if study is conducted in three different settings.

Table 2. Sample size assumptions for a clinical trial of corticosteroids for AOM conducted in different settings.

Proportion of children	Original assumption [7]*	Middle scenario	Pilot observed result†
With severe AOM	35%	56%	78%
With severe AOM AND ongoing pain	57.5%	50%	42%
With mild AOM	65%	43%	22%
With mild AOM AND ongoing pain	36%	46%	57%
With severe and mild AOM AND ongoing pain	31.6%	38.4%	45.2%
Sample size calculation‡	760	570	444

*From a meta-analysis of studies conducted in developed countries; †Our pilot study was conducted in a developing country, urban setting; ‡The sample size includes a 10% allowance for dropouts.

3.12 Recruitment and stratification

Prior to the study, we will provide training and study procedure manuals for the participating healthcare personnel (i.e. GPs, ENT specialists, nurses, pharmacists) which describe all processes and procedures for recruitment, stratification, outcome measures, and randomisation, including data collection and recording using CRFs and the symptom diary. We will also detail the severity criteria for AOM and instructions for clinical assessment using an otoscope. We expect the participating

healthcare personnel will be able to effectively and sufficiently conduct these study procedures and measures. We will allocate research assistants to assist the study recruitment and randomisation at study sites.

We will recruit and stratify children based on the eligibility and stratification criteria. Since we will involve both GPs and the ENT specialists at the primary care centres and hospitals, we will stratify children based on healthcare centre levels (primary versus secondary/tertiary care centres) and AOM severity (mild versus severe AOM). Children will be stratified into the severe group if they have at least one of the following criteria: (1) moderate or severe symptoms of AOM, locally (e.g. moderate or severe ear pain, mastoiditis), and/or systemically (e.g. fever with temperature 39°C or higher, irritable, vomiting); (2) moderate or severe signs of middle ear inflammation (e.g. hyperaemic tympanic membrane) and effusion (e.g. bulging, bulla formation, yellowish purulent appearance of tympanic membrane); (3) aged younger than 2 years with bilateral AOM; or (4) tympanic membrane perforation (see Figure 1).

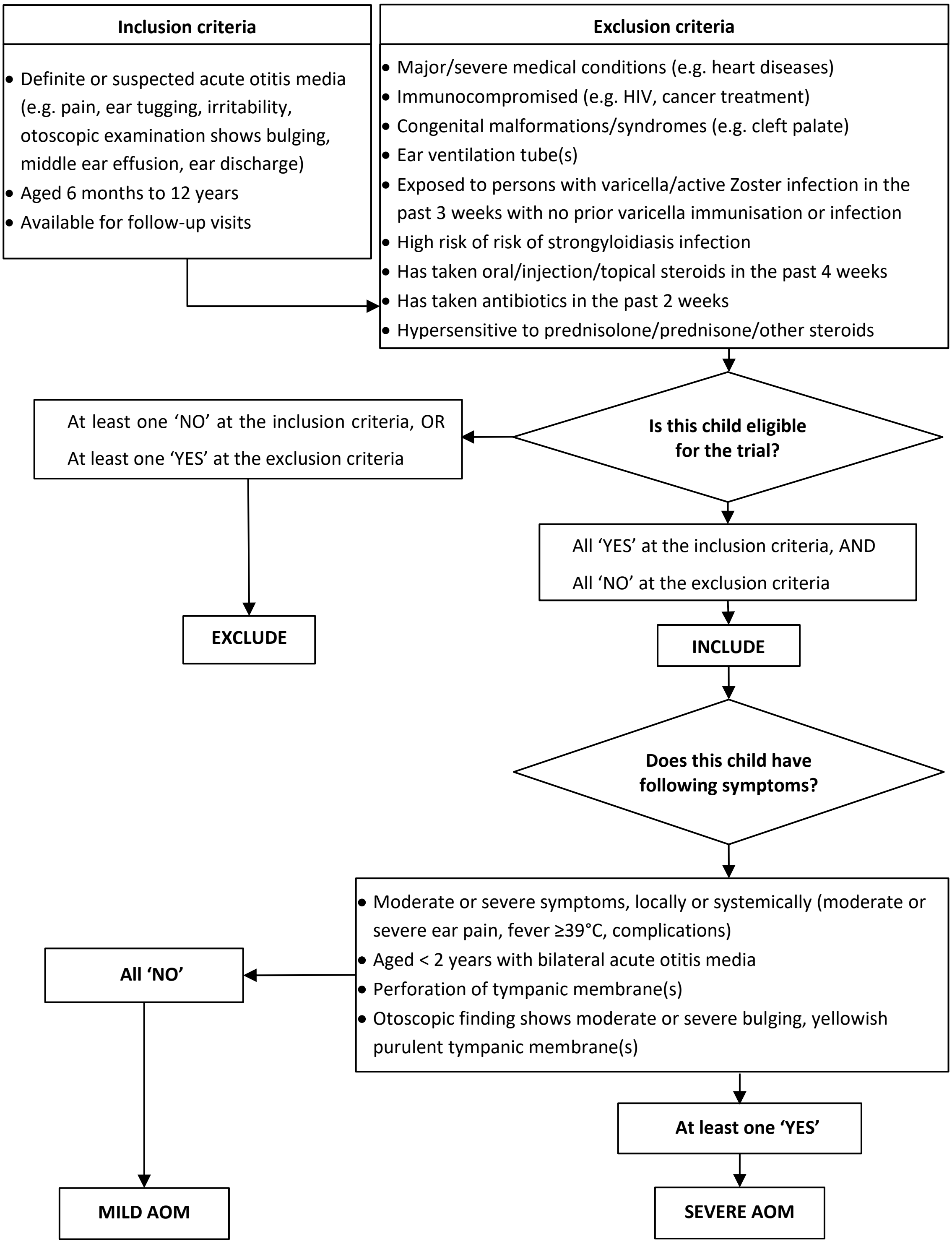

Figure 1. Eligibility and stratification diagram.

3.13 Randomisation and allocation concealment

All consenting eligible children and their parents will be enrolled and stratified based on healthcare service level and AOM severity (see Figure 2). The physician will record the recruitment and the

stratification in the eligibility form, whilst the nurse will later collect and record personal information of the study participants and their parents (e.g. date of birth, mobile phone numbers, home address) in the study registration form. Each study participant will be identified by a 3-digit study ID, which will be written on every CRF. The nurse will use the eligibility and study registration forms for randomisation purposes. We will provide two options to access the study randomisation: either the nurse can access MASCoT (an online randomisation generator developed by the Institute for Evidence-Based Healthcare (IEBH), Bond University, Queensland, Australia), or contact our 24-hour call centre, if using MASCoT is not possible. A successful randomisation will provide a 3-digit randomisation ID, which will be recorded in the randomisation form and study prescription. A permuted block randomisation sequence will be computer-generated by MASCoT, prior to study commencement. The allocation sequence will remain concealed throughout the study. No one but the statistician, who is blinded to study condition and concurrent medication, has access to MASCoT except for any emergency cases requiring unblinding. The children will then be randomly allocated to either prednisolone plus expectant observation, or placebo plus expectant observation alone in the mild group; and either prednisolone plus antibiotic, or placebo plus antibiotic in the severe group.

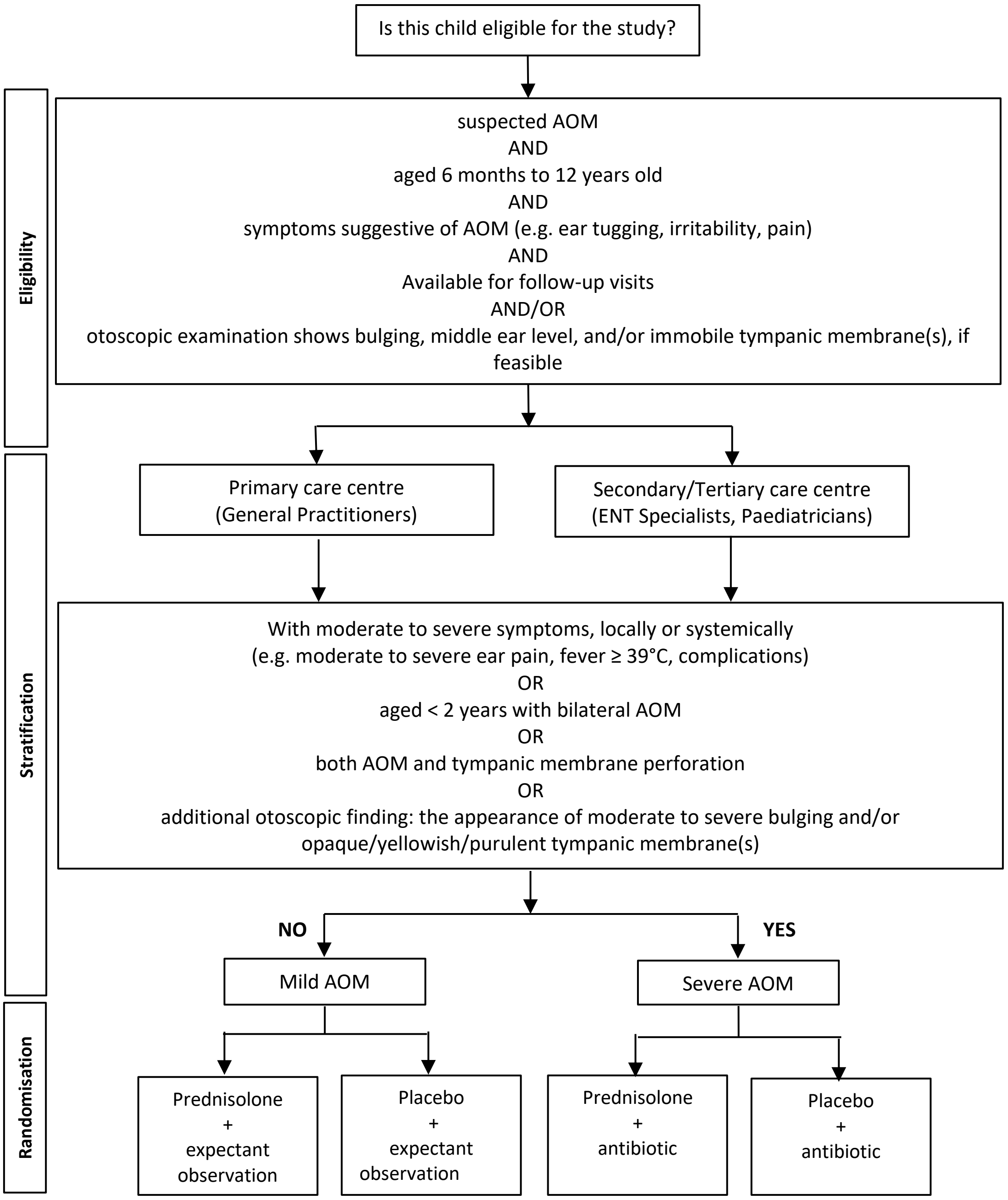

Figure 2. Flow chart of the stratification and randomization of the study

During the consultation, the physician will dispense two prescriptions to the nurse. The first prescription is for concurrent medications (e.g. antibiotics for severe AOM, analgesics, decongestants); whilst the second prescription is for study medication. The second prescription will provide clear information on the dose of study medication (based on the age), date of birth (to confirm the dose), and registration ID (for the allocation of the study medication). Randomisation ID will be added after the randomisation process by the study nurse.

Using the information in the prescription, the pharmacist will prepare the study medication, either the prednisolone or placebo, by crushing the tablets based on the prescribed dose and mixing them with sweeteners, then pack the mixed powder in five daily paper wraps. The pharmacist will then dispense the study medication along with instructions for the parents, and record the dispensing on the form provided by the study for this purpose. Batches of study medication will be dispatched to participating healthcare centres from a central pharmacy facility at the Clinical Research Supporting Unit, Faculty of Medicine Universitas Indonesia (CRSU FMUI).

3.14 Blinding

The physicians, nurses, pharmacists, research assistants, and both study participants and their parents will be blinded to the intervention allocation throughout the study. Emergency unblinding can occur if there are serious adverse events (SAEs). The unblinding result will be limited only to the treating physician, the parent/caregiver of the study participant who is experiencing the SAE, the study statistician, and the principal investigator.

3.15 Data collection methods

We will collect all data and outcome using CRFs (i.e. consent form, eligibility form, outcomes form, randomisation form, serious adverse effects form) and a symptom diary.

To assess the proportion of children with ongoing pain (VAS score of ≥ 5 mm) and reduction of pain intensity at various timepoints, we will use the VAS score in the symptom diary which will be completed by the parents or older capable study participants. The VAS is a well-established and validated scale for pain assessment and commonly used for research [13]. This 100-mm horizontal pain scale has a 'no pain' anchor at the left and a 'the most severe pain' at the right endpoint. We will measure the distance between the left endpoint to a vertical line crossing the horizontal scale which represents the intensity of the pain. This vertical line will be marked by the parents or study participants aged eight years or older who are capable of self-pain measurement [14]. We will identify whether the reduction of pain intensity is clinically meaningful by using VAS score of 10 mm as a minimum clinically important difference between groups [15,16].

To assess the reduction of overall AOM-relevant symptoms, we will use the AOM-SOS that will be completed by the parents in the symptom diary. We will use AOM–SOS to identify other symptoms that are commonly found in young children with AOM [17]. The scale consists of three possible intensities of symptoms, which are 'no' (0 point), 'a little' (1 point), and 'a lot' (2 points), Table 3. The

minimum clinically important difference between groups for total AOM-SOS points is 4.2 points [17]. Prior to the pilot study, we translated the AOM-SOS to the Indonesian language [12].

Table 3. Acute otitis media severity of symptoms scale (AOM-SOS) [17]

**We are interested in finding out how your child has been doing. For each question, please place a check mark in the box corresponding to your child's symptoms. Please answer all questions.**			
	**No**	**A little**	**A lot**
Over the past 12 hours, has your child been tugging, rubbing, or holding the ear(s) more than usual?	☐	☐	☐
Over the past 12 hours, has your child been crying more than usual?	☐	☐	☐
Over the past 12 hours, has your child been more irritable or fussy than usual?	☐	☐	☐
Over the past 12 hours, has your child been having more difficulty sleeping than usual?	☐	☐	☐
Over the past 12 hours, has your child been less playful or active than usual?	☐	☐	☐
Over the past 12 hours, has your child been eating less than usual?	☐	☐	☐
Over the past 12 hours, has your child been having fever or feeling warm to touch?	☐	☐	☐

To assess the need for antibiotics, we will use the outcome form completed by participating clinicians and the symptom diary completed by the parents to identify any antibiotic prescribing from other physicians for AOM or other health issues. We will identify any antibiotic initiation for children in the mild group or second line antibiotics for children in the severe group during the first 2 weeks of the study.

To assess complications and adverse events, we will also use the outcome form and the symptom diary. In terms of adverse events, we will provide several common side effects related to corticosteroid use in the symptom diary. We will also ask parents to record any other side effects that are not listed in the symptom diary.

To assess AOM recurrence, we will interview the parents by phone at day 30 and day 90. We will identify any new episode of AOM and record this in the outcome form.

To promote study retention and complete follow-up, we will send a daily text message reminder to the parents during the first two weeks. This message will remind the parents to give the study medication to their children for 5 days and to complete the symptom diary for 2 weeks. We will remind the parents to take their children for the follow-up visits at day 3 and day 7. We will confirm their visit 1 to 3 days prior to the scheduled time, which allows us to organise another time or home visit if they are not able to come. If the study participants are not able to come at the scheduled follow-up time, we will encourage them to come up to maximum of 3 days over the scheduled time. We will provide an incentive in the form of reimbursement for their transportation to and from the primary care centres/hospitals and a small souvenir for study participants. We will advise the parents to take their children to the primary care centres/hospitals if there are any concerning conditions or no improvement. We will record any cases of discontinuation or deviation from study protocol in CRFs and continually follow these study participants per protocol where possible.

3.16 Data management

We will consistently conduct cross-checks during and after data entry to maintain the integrity and completion of data, particularly for missing data or errors. We will document all data entry and modifications in the database which will be available for viewing. Any modifications to the study data will be completed with the name, date, and signature of the person who is responsible for data modification. For missing or erroneous data, we will confirm the study data in the database using the original CRFs or by sending queries to the originating site. The central data coordinator will check the validity and completeness of study data on a regular basis. All data in the central database will be protected with a regular complete back up system.

3.17 Statistical methods

For dichotomous outcomes (i.e. the proportion of children with ongoing pain at various time points, who require antibiotic initiation in mild group or second-line antibiotics treatment for children with severe group after 48-hour observation, with complications related to AOM, and with AOM recurrence), we will conduct a chi-squared test to determine the differences between two groups in outcome, expressed as relative risk (RR) with 95% confidence intervals (CI) and p values. A secondary analysis will use multiple logistic regression to adjust the primary outcome for allergy or atopy, siblings, first episode of AOM before the age of 12 months, passive smoking, breastfeeding duration less than three months, low parental education level, and day care attendance.

We will report the proportion of children with adverse events and children who are adherent to the study and study medication in percentages.

For continuous outcomes of reduction of pain intensity measured using VAS and overall AOM-relevant symptoms measured using AOM-SOS, we will conduct independent t-tests to determine the differences between two groups in mean score, expressed as mean difference (MD) with 95% CIs and *p* values. We will also conduct multiple linear regression, adjusting for similar factors as the multiple logistic regression above (equivalent to ANCOVA). For outcome of time duration to pain resolution, we will use a log rank test to compare the time to pain resolution between the two groups. We will use Kaplan-Meier survival plots to show the proportion of children who experienced the outcomes at each time point. We will use STATA 15.1 software for statistical analysis.

We plan to analyse by intention-to-treat; however, if there is loss to follow-up, we will use an available case analysis. We will still record data on study participants who stop study medication, where possible, and will include them in the analysis where outcome data are available.

4. Data monitoring

An independent person from Clinical Epidemiology and Evidence-Based Medicine (CEEBM) Unit, Dr. Cipto Mangunkusumo Hospital (CMH) – Faculty of Medicine Universitas Indonesia (FMUI) will perform data monitoring periodically. She will review the process of patient recruitment, data entry, and study data storage in the central database, and report the results to the steering committee and sponsor as to whether the research has been conducted appropriately based on the approved research protocol.

We will perform an interim-analysis when 50% of patients have been randomised and have completed the 3-month follow-up. The interim analysis will be conducted by independent statistician from CRSU FMUI, who will be blinded to the treatment allocation and will report to a data and safety monitoring committee (DSMC) in CRSU FMUI. The DSMC will receive access to unblinded data of the trial if requested and will make recommendations to the steering committee without revealing specific details of the results unless there is a strong recommendation to cease the study. The decision on the continuation of the trial will be decided by the steering committee based on the recommendation from the DSMC.

5. Harm

We define adverse events (AEs) as 'any untoward medical occurrence in a patient or clinical investigation subject administered a pharmaceutical product and which does not necessarily have to

have a causal relationship with study medication', whereas adverse effects or adverse drug reactions (ADRs) are defined as 'all noxious and unintended responses to a study medication related to any dose' [12]. Data collection of any AEs and ADRs commences after we obtain consent from the parents and enrol the eligible children into the study. We will record all AEs or/and ADRs following the enrolment during the study in the outcome form and serious adverse effects form. We will also use an adverse event assessment form which will be completed by an expert team established for this purpose. The expert team includes paediatricians and ENT specialists who have experience in conducting a clinical trial and are competent in the clinical fields of paediatric inflammatory disorders.

We define serious adverse events as 'any untoward medical occurrence at any dose that may result in-patient and/or prolonged hospitalisation, persistent or significant disability, medically important events, life-threatening events, and death' [12]. A detailed information of the management of serious adverse events in the study can be retrieved from our pilot study protocol [12].

6. Auditing

We will establish an independent audit committee from the CRSU FMUI and CEEBM Unit CMH-FMUI. This independent committee will conduct monitoring of source paper and electronic documents in the electronic database, monitor the conduct of trial in multicentre sites, interview the investigators and coordinators, and check the storage, distribution, and the use of study medication. Before the commencement of the study, the committee will ensure that the research staff are competent in data entry and in using the electronic database. These processes will be conducted according to the protocol and International Conference Harmonization – Good Clinical Practice (ICH-GCP) standards. The data monitoring is scheduled to be conducted every six months from the research head office (CEEBM Unit CMH-FMUI) and trial sites.

7. Ethics and dissemination

Research ethics approval

This study will be conducted according to the Declaration of Helsinki and ICH-GCP guidelines. We will obtain research ethics approval from the Bond University's Human Research Ethics Committee (BUHREC) Australia and the Ethics Committee FMUI Indonesia. We will also seek permits to conduct clinical research from the One Stop Integrated Service Agency, Province of DKI Jakarta and from each study site.

Protocol amendments

Any modifications to the protocol which may impact on the trial process (e.g. the modification of study objectives, study design or/and procedures, study population, sample sizes), potential benefits and safety of the study participants will require a formal amendment to the protocol. This amendment will be sent to and approved by the funding body and the Ethics Committee prior to its implementation. Notification will also be sent to the health authorities in accordance with local regulations. Minor modifications that may not impact on the trial process will also be notified and approved by the funding body and will be notified to the Ethics Committee.

Consent or assent

Before obtaining consent for their participation in the study, we will provide structured and detailed study information, such as the justification for the study, all study procedures, and expected commitment of the parents/caregivers during the study (e.g. give the study medication, complete the symptom diary, and come to follow-up visits per protocol), as well as potential side effects from the study medication. We will obtain the consent of study participation from children aged 12 years and over. The person who delivers the consent interview will also provide their signature on the consent form, stating that they have provided information and opportunity for potential participants to understand and raise relevant questions about the study. We will ensure the consent process is free of coercion, and that parents understand their right to withdraw from the study at any time without any consequences on healthcare services, as participation is voluntary.

Confidentiality

The original CRFs and other study information will be stored in a secure locked file cabinet at each participating site, with copies at the central research office. All study information stored in the study database will be secured with password-protected systems under limited access. We will use a 3-digit study ID as an identifier for CRFs and other forms related to the study. The study data storage will separate forms containing names (i.e. consent form, study registration form) from CRFs and other study forms. All counselling sessions will be conducted in private rooms. We will ask all participating healthcare personnel to maintain the confidentiality of study participants.

Data on every participant will be kept confidentially and will not be distributed externally without the written permission of the participant, except if it is required for trial monitoring by national regulatory authorities related to medical and research safety.

8. Access to data

The principal investigator (RR) will be given access to the cleaned data sets. RR will also have direct access to each site's data sets on request. Project data sets will be secured using passwords. To ensure confidentiality, data dispersed to project team members will be blinded of any identifying participant information. We will make our cleaned study data publicly available in the Bond University research repository (https://research.bond.edu.au/) after the completion of the study and the publication of the study results.

9. Ancillary and post-trial care

Any complications of AOM and potential adverse effects from the study will be closely monitored using the symptom diary. In case there is an emergency or worsening condition that requires comprehensive assessment and management, we will provide a 24-hour call centre and list of phone numbers and addresses of healthcare providers. We will be responsible for the treatment of any adverse effects that occur from the study medication during the study. The compensation will include costs such as consultation visits, additional examinations, and treatment (e.g. medicine, hospitalisation cost). Due to other potential concurrent treatments within the study, there will be robust review and analysis to ascertain the cause of adverse events. Information on management of adverse effects will be provided by physicians during the consent approval process before entering the study. We will also include this information in the patient symptom diary. We will provide contact details of the 24-hour call centre and a list of recommended healthcare providers on the follow-up card.

10. Dissemination policy

We will report the study results in a medical journal and by presentations at conferences or other medical meetings. All investigators will review the manuscript and provide their consent for their acknowledgment and contribution before submission.

References

1. Lieberthal AS, Carroll AE, Chonmaitree T, Ganiats TG, Hoberman A, Jackson MA, et al. Clinical practice guideline: the diagnosis and management of acute otitis media. Pediatrics. 2013;131:e964–e99.
2. Liese JG, Silfverdal SA, Giaquinto C, Carmona A, Larcombe JH, Garcia-Sicilia J, et al. Incidence and clinical presentation of acute otitis media in children aged <6 years in European medical practices. Epidemiol Infect. 2014;142:1778–88.
3. South Australian Child Health Clinical Network. Australian Policy Clinical Guideline South Australian Paediatric Practice Guidelines: Acute Otitis Media in Children. https://www.sahealth.sa.gov.au/wps/wcm/connect/a8910c004329b4dc81b8ed8bf287c74e/Acute+Otitis+Media+in+children_May2014.pdf?MOD=AJPERES&CACHEID=a8910c004329b4dc81b8ed8bf287c74e. Published February 2014. Accessed February 22, 2016.
4. McCullough AR, Pollack AJ, Hansen MP, Glasziou PP, Looke DFM, Britt HC, et al. Antibiotics for acute respiratory infections in general practice: comparison of prescribing rates with guideline recommendations. Med J Aust. 2017;207(2):65–9.
5. Henderson J, Valenti L, Miller GC. General practice antibiotic prescribing for management of otitis media in children. Aust Fam Physician. 2016;45(6):363-5.
6. Le Saux N, Robinson JL, Canadian Paediatric Society Infectious Diseases and Immunization Committee. Management of acute otitis media in children six months of age and older. Paediatr Child Health. 2016;21(1):39-44.
7. Rovers MM, Glasziou P, Appelman CL, Burke P, McCormick DP, Damoiseaux RA, et al. Antibiotics for acute otitis media: a meta-analysis with individual patient data. Lancet. 2006;368:1429-1435.
8. Ranakusuma RW, Pitoyo Y, Safitri ED, Thorning S, Beller EM, Sastroasmoro S, Del Mar CB. Systemic corticosteroids for acute otitis media in children. Cochrane Database Syst Rev. 2018;3:CD012289. https://doi.org/10.1002/14651858.CD012289.pub2.
9. Ruohola A, Heikkinen T, Jero J, Puhakka T, Juvén T, Närkiö-Mäkelä M, et al. Oral prednisolone is an effective adjuvant therapy for acute otitis media with discharge through tympanostomy tubes. J Pediatr. 1999;134:459–63.
10. Francis NA, Cannings-John R, Waldron CA, Thomas-Jones E, Winfield T, Shepherd V, et al. Oral steroids for resolution of otitis media with effusion in children (OSTRICH): a double-blinded, placebo-controlled randomised trial. Lancet. 2018;392:557-68.
11. Jensen MP, Chen C, Brugger AM. Interpretation of visual analog scale ratings and change scores: a reanalysis of two clinical trials of postoperative pain. J Pain. 2003 Sep;4(7):407-14

12. Ranakusuma WR, McCullough AR, Safitri ED, Pitoyo Y, Widyaningsih, Del Mar CB, et al. Oral prednisolone for acute otitis media in children: protocol of a pilot randomised, open-label, controlled study (OPAL study). Pilot and Feasibility Studies. 2018;4:146. https://doi.org/10.1186/s40814-018-0337-x.
13. Huguet A, Stinson JN, McGrath PJ. Measurement of self-reported pain intensity in children and adolescents. J Psychosom Res. 2010;68:329–36.
14. Cohen LL, Lemanek K, Blount RL, et al. Evidence-based assessment of paediatric pain. J Pediatr Psychol. 2008;33(9):939–56.
15. Von Baeyer C. Children's self-report of pain intensity: what we know, where we are headed. Pain Res Manag. 2009;14(1):39–45.
16. Powell CV, Kelly AM, Williams A. Determining the minimum clinically significant difference in visual analogue pain score for children. Ann Emerg Med. 2001;37(1):28–31.
17. Shaikh N, Hoberman A, Paradise JL, et al. Responsiveness and construct validity of a symptom scale for acute otitis media. Pediatr Infect Dis J. 2009;28(1):9–12.
